# A Bilayer Rare‐Earth/High‐κ Oxide Memristor for Energy‐Efficient Neuromorphic Intelligence

**DOI:** 10.1002/smll.73836

**Published:** 2026-05-20

**Authors:** Hammad Ghazanfar, Muhammad Rabeel, Honggyun Kim, Sobia Nisar, Muhammad Wajid Zulfiqar, Muneeb Ahmad, Rana Faryad Ali, Ghulam Dastgeer, Deok‐kee Kim

**Affiliations:** ^1^ Department of Semiconductor Systems Engineering Sejong University Seoul Republic of Korea; ^2^ Department of Electrical Engineering and Convergence Engineering for Intelligent Drone Sejong University Seoul Republic of Korea; ^3^ Department of Optical Engineering Sejong University Seoul Republic of Korea; ^4^ Department of Materials Science and Engineering and Materials Research Laboratory Massachusetts Institute of Technology (MIT) Cambridge Massachusetts USA; ^5^ Department of Physics and Astronomy Sejong University Seoul Republic of Korea

**Keywords:** artificial synapse, edge AI, interface‐engineering, memristor, neuromorphic computing, rare‐earth

## Abstract

The growing demand for brain‐inspired computing systems has intensified research into energy‐efficient, scalable, and adaptive hardware that mimics biological synaptic behavior. Neuromorphic memristor devices, which integrate memory and processing functionalities within a single nanoscale unit, are emerging as promising building blocks for next‐generation artificial intelligence systems. In this work, we demonstrate a CMOS‐compatible Ag/Gd_2_O_3_/HfO_2_/Pt bilayer memristor engineered with atomically sharp interfaces and optimized defect landscapes to achieve stable and efficient resistive switching behavior. The device exhibits excellent performance, including an ON/OFF current ratio exceeding 10^7^, retention beyond 10^4^ s, a sub‐microsecond switching transition time (350 ns), and low programming energy of just 13.6 pJ. Interface engineering effectively stabilizes multilevel conductance states, suppresses stochastic filament growth, and supports analog long‐term potentiation and depression. Incorporating the experimentally measured synaptic plasticity into convolutional neural network simulations yields a 78% classification accuracy on the Fashion‐MNIST dataset, along with robust color recognition. These results demonstrate, as a device‐level proof of concept, that bilayer rare‐earth/high‐κ oxide memristors can inform the development of future non‐volatile memory and low‐power edge neuromorphic systems.

## Graphical abstract

Interface‐engineered Gd_2_O_3_/HfO_2_ bilayer memristors demonstrate controlled filament formation, ultralow switching energy (∼13.56 pJ), and fast operation (∼350 ns) with a high ON/OFF ratio (∼10^7^). The devices exhibit stable analog synaptic behavior and enable pattern recognition on Fashion‐MNIST, underscoring their promise for energy‐efficient neuromorphic computing and edge AI hardware applications.

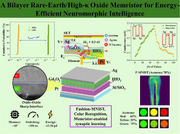

## 1 Introduction

Artificial intelligence (AI) and neuromorphic computing are approaching their limits due to energy inefficiency and latency issues in traditional von Neumann architectures, prompting the search for new hardware paradigms that combine memory and computation [1, 2, 3, 4]. The increasing data demands of these workloads heavily impact memory performance, endurance, and efficiency, particularly in edge devices, mobile platforms, and wearables, which are often limited by power constraints. Conventional volatile memories like static random‐access memory (SRAM) and dynamic random‐access memory (DRAM) experience both dynamic and static power loss, making them suboptimal for large‐scale, energy‐efficient AI systems [5, 6]. While flash memory provides non‐volatility, its relatively slow programming speed, typically >10 µs, and limited endurance restrict its suitability for high‐speed data‐intensive AI processing [7, 8, 9]. Recent studies have explored metal‐oxide resistive switching memristors for neuromorphic applications, demonstrating their ability to emulate synaptic behavior with high energy efficiency and compatibility with CMOS processes [10, 11, 12, 13, 14].

While single‐layer oxide memristors have garnered significant attention for their simplicity, they frequently exhibit drawbacks such as stochastic switching, excessive device‐to‐device variability, and poor control over multi‐level conductance due to the random nature of filament formation within a uniform matrix [15, 16, 17]. In contrast, recent studies have shown that bilayer or multilayer memristor structures can significantly enhance device reliability, retention, and analog tunability by introducing a well‐defined interface between two dissimilar oxide materials [18, 19, 20]. This engineered interface can modulate the local electric field, control the spatial distribution of oxygen vacancies, and provide an additional energy barrier for ion migration, thereby facilitating more reproducible filament nucleation and gradual conductance modulation. For instance, bilayer oxide memristors have demonstrated switching exceeding 10^6^ cycles and an ON/OFF ratio above 10^8^, in contrast to 10^3^–10^4^ cycles and lower ON/OFF ratios typically observed in single‐layer devices [21, 22, 23]. These advances underline the critical role of the material interface in achieving the robust, analog switching characteristics required for next‐generation neuromorphic hardware. Despite these advances, further innovations in material engineering and device architecture are required to realize robust, high‐speed, and multilevel memristive devices suitable for future neuromorphic system integration [11, 24, 25].

Rare‐earth oxides such as gadolinium oxides (Gd_2_O_3_) and high‐к dielectrics such as hafnium oxide (HfO_2_) have emerged as leading candidates for resistive switching and neuromorphic device applications due to their unique physical and electrical properties [18, 26]. HfO_2_ is widely recognized for its high dielectric constant ∼25, wide bandgap ∼5.6 eV, excellent thermal stability, and established compatibility with CMOS fabrication, making it a foundational material for scalable memristive architectures [27, 28]. Gd_2_O_3_, with a dielectric constant of ∼14 and a wide bandgap of ∼5.3 eV, offers low leakage current, a strong tendency for oxygen vacancy formation (oxygen vacancy formation energy ∼4.1 eV), and robust chemical stability, collectively enabling stable switching behavior essential for synaptic emulation [29, 30, 31]. Both oxides allow controlled migration of oxygen vacancies and metal ions under an electric field, with a reported ion migration activation energy of 0.8–1.2 eV, underpinning reliable and repeatable filamentary switching. In this work, we present a CMOS‐compatible bilayer Ag/Gd_2_O_3_/HfO_2_/Pt memristor featuring atomically sharp oxide‐oxide interfaces engineered for robust, resistive switching and neuromorphic hardware integration. The device achieves reliable, non‐volatile memory operation with an ON/OFF current ratio of ∼10^7^, stable endurance over 100 cycles, and excellent retention at 10,000 s. Notably, the system demonstrates rapid switching dynamics, with a minimum switching time of 350 ns and low programming energy of 13.56 pJ (as calculated in Figure S6). Multi‐level conductance states are accessible via pulse engineering, enabling both digital and analog memory functionality. Integration of the measured device plasticity directly into a convolutional neural network (CNN) enables hardware‐constrained inference in simulation for both pattern and color recognition, while explicitly reflecting analog variability. Collectively, these advances highlight the promise of interface‐engineered rare‐earth/high‐k oxide bilayer memristors as scalable, energy‐efficient synaptic elements for next‐generation neuromorphic and edge‐AI. This work is presented as a device‐level proof‐of‐concept focused on the bilayer interface and measured synaptic behavior. Array‐level demonstrations and circuit benchmarking are beyond the present scope and are outlined as future directions.

## 2 Results and Discussion

### 2.1 Structural Characterization

**FIGURE 1:**
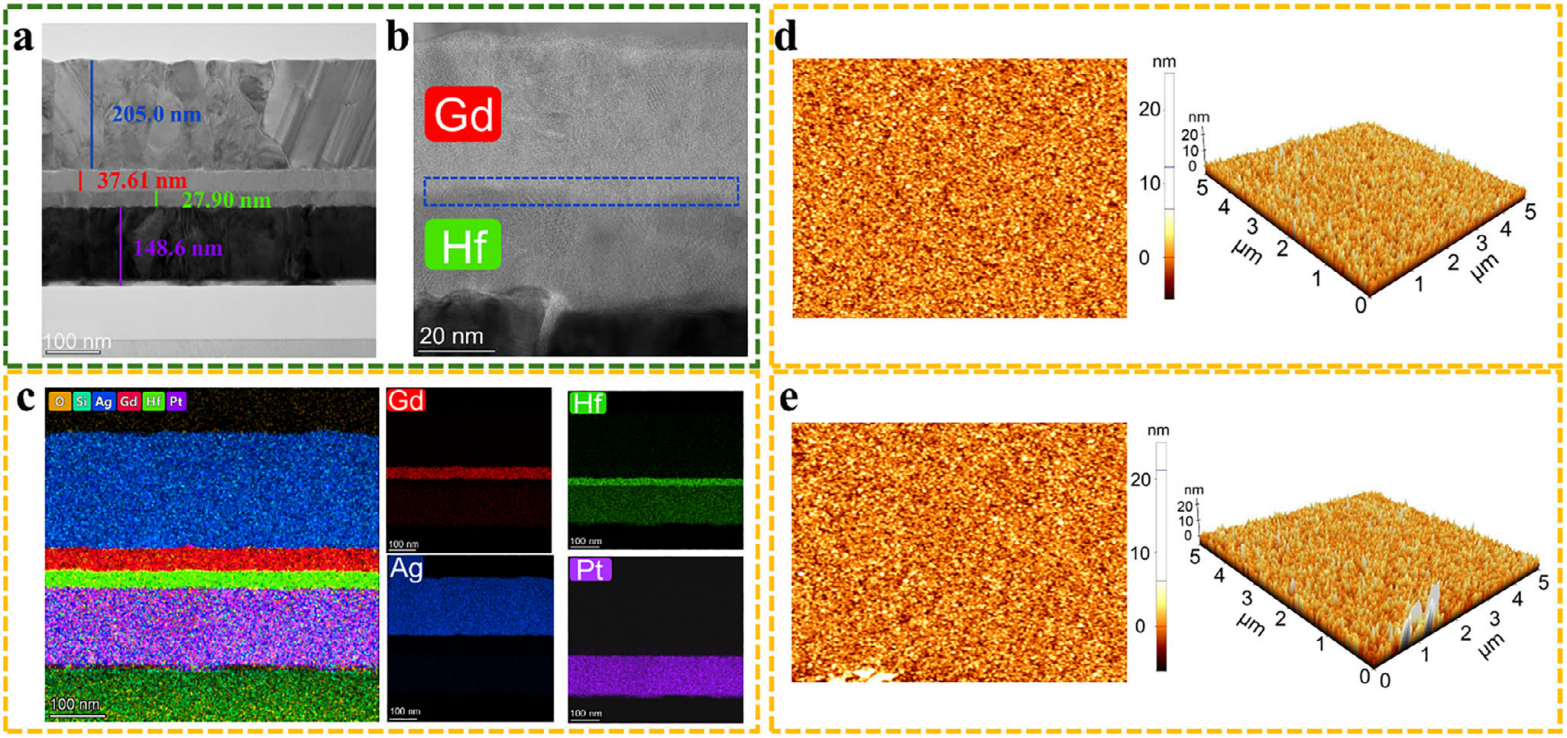
Structural and morphological characterization of the Ag/Gd_2_O_3_/HfO_2_/Pt memristor device. (a) Cross‐sectional TEM image showing the full device stack with clearly resolved Ag (205 nm), Gd_2_O_3_ (37.61nm), HfO_2_ (27.90 nm), and Pt (148.6 nm) layers. (b) High‐resolution TEM (HRTEM) image of the Gd_2_O_3_/HfO_2_ interface, highlighting sharp, well‐defined boundaries and uniform thickness. (c) EDS elemental mapping of the device cross‐section, including individual maps for Ag, Gd, Hf, and Pt, confirms uniform elemental distribution and clear separation between layers. (d) Atomic force microscopy (AFM) images of the device surface after annealing, shown in 2D and 3D display a smoother, homogeneous surface, indicating minor morphological changes post‐annealing. (e) AFM images before annealing, shown in 2D and 3D, reveal a slightly high but uniform surface roughness, a homogeneous surface, confirming high‐quality film formation before thermal treatment. These characterizations demonstrate the successful fabrication of the multilayer memristor, with well‐defined, homogeneous elemental composition, and controlled surface roughness suitable for reliable device operation.

**FIGURE 2:**
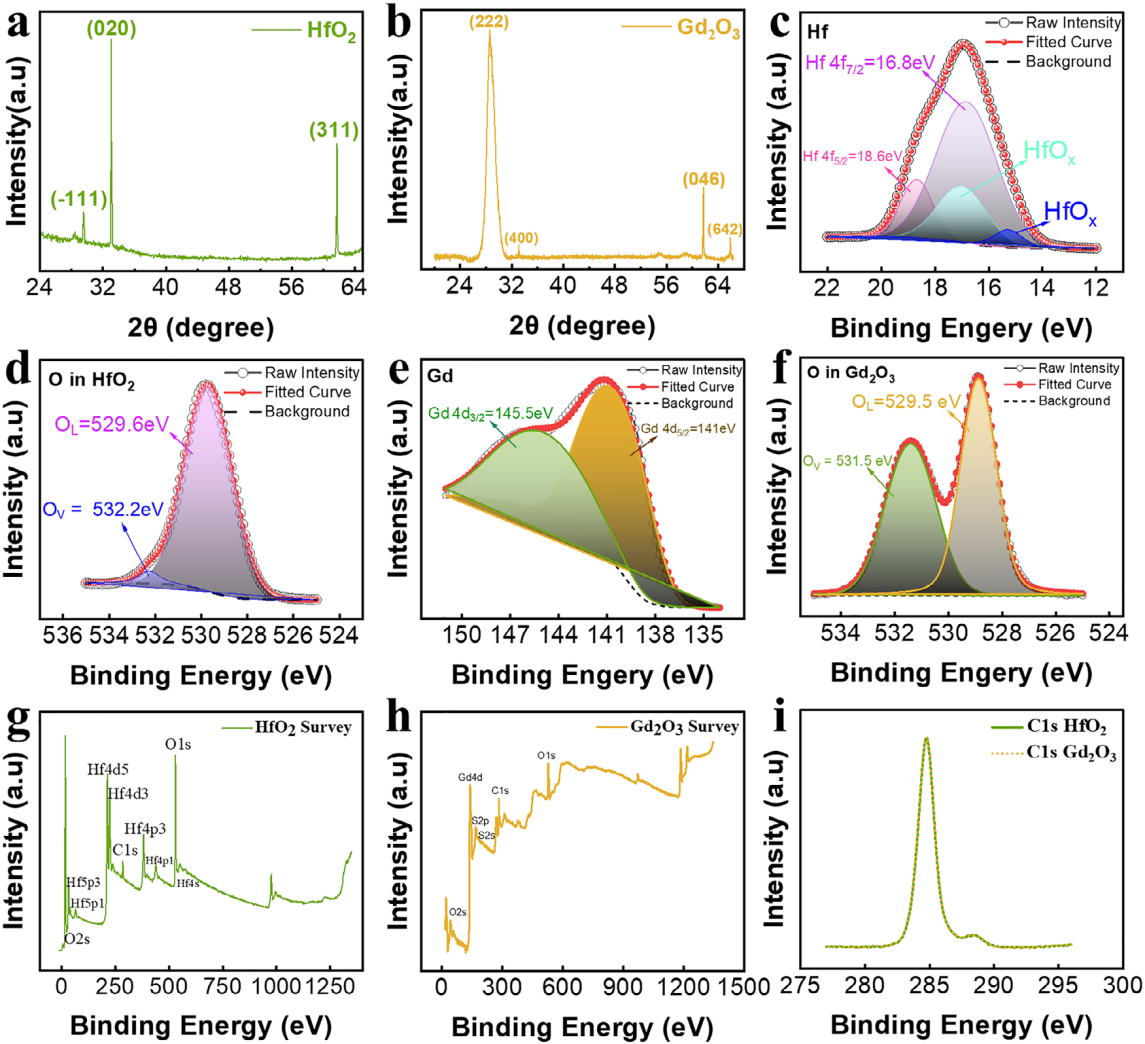
Structural and chemical analysis of HfO_2_ and Gd_2_O_3_ thin films in the memristor device. (a) X‐ray diffraction (XRD) pattern of ALD‐grown HfO_2_, with peaks at 2θ = 28.3°, 31.6°, and 54.4° corresponding to the (−111),(020), and (311) planes of monoclinic HfO_2_ (JCPDS # 34–104). (b) XRD pattern of the sputtered Gd_2_O_3_ film, displaying peaks at 2θ = 29.1°, 33.1°, 56.2°, and 65.8°, which match the (222), (400), (440), and (622) planes of the cubic phase (JCPDS # 00‐012‐0797). (c) High‐resolution XPS spectrum of Hf4f in HfO_2_, showing peaks at 16.8 eV (Hf 4f_7/2_) and 18.6 eV (Hf 4f_5/2_) attributed to fully oxidized Hf^4+^, with an additional sub‐peak near 17–18 eV assigned to HfO_x_, indicating oxygen‐deficient, non‐stoichometric Hf region. (d) XPS O 1s spectrum in HfO_2_, with a primary peak at 529.6 eV due to lattice oxygen (O_L_) and a secondary peak at 532.2 eV attributed to oxygen vacancies (O_v_). (e) XPS Gd 4d spectrum in Gd_2_O_3_, showing main peaks at 145.5 eV (Gd 4d_5/2_) and 141.0 eV (Gd 4d_3/2_), confirming the presence of the Gd^3+^ oxidation state. (f) XPS O 1s spectrum in Gd_2_O_3_, with a peak at 529.5 eV for lattice oxygen (O_L_) and a second peak at 531.5 eV for oxygen‐deficient (O_v_) regions. (g) XPS survey spectrum for the HfO_2_ layer, confirming strong Hf and O signals without significant impurities. (h) XPS survey spectrum for the Gd_2_O_3_ layer, showing clear Gd and O signatures. (i) XPS C 1s spectrum, revealing minor surface contamination at 284.8 eV on both HfO_2_ and Gd_2_O_3_ films. These results confirm the phase purity, chemical states, and presence of oxygen vacancies in HfO_2_ and Gd_2_O_3_ films, which are essential for reliable resistive switching and device performance.

The structural quality and interface sharpness of the bilayer Ag/Gd_2_O_3_/HfO_2_/Pt memristor stack were first evaluated using a combination of high‐resolution cross‐sectional transmission electron microscopy (HR‐TEM), energy‐dispersive x‐ray spectroscopy (EDS), atomic force microscopy (AFM), and x‐ray diffraction (XRD), as shown in Figures 1 and 2. The device fabrication employed atomic layer deposition (ALD) for HfO_2_ and RF sputtering for Gd_2_O_3_, followed by heat treatment at 500°C for 2 h in an argon atmosphere to optimize crystallinity and interface uniformity. The cross‐sectional TEM image, as shown in Figure 1a, reveals a distinctly stratified Ag/Gd_2_O_3_/HfO_2_/Pt structure, with the Gd_2_O3 and HfO_2_ films exhibiting uniform thicknesses of 37.61 and 27.90 nm, respectively. Notably, the interfaces between the oxide layers are atomically abrupt and free from observable intermixing or secondary phase formation, which is a direct consequence of carefully optimized sequential deposition and controlled thermal budget. Such interface sharpness plays a critical role in bilayer memristors, as it enables modulation of the local electric field and defect distribution at the oxide‐oxide boundary, which influences oxygen vacancy migration and filament nucleation dynamics. By constraining the filament formation region to a sharply defined interface, the device is expected to exhibit reduced stochasticity and improved cycle‐to‐cycle reproducibility consistent with theoretical and experimental reports [18]. High‐resolution TEM in Figure 1b further demonstrates the crystalline order of both Gd_2_O_3_ and HfO_2_ layers, with well‐resolved lattice fringes extending up to the interface. This crystallinity is not only indicative of high‐quality film growth but is also functionally relevant; crystalline oxides possess lower intrinsic defect densities and more predictable ion migration pathways than their amorphous counterparts, enabling sharper SET/RESET transitions and improved modulation in resistive switching devices. The sharp interface, as observed, can introduce a heterojunction‐like effect, altering the local potential landscape and selectively filtering ion migration, a mechanism that has been shown to suppress undesired filament overgrowth and thus expand the attainable window of conductance states [32].

Elemental mapping via EDS, as shown in Figure 1c, along with corresponding elemental panels, confirms the spatial segregation of Ag (top electrode), Gd, Hf, Pt (bottom electrode), and O within their respective layers, with minimal interdiffusion. This chemical fidelity not only validates the fabrication process but also ensures that the switching behavior is strongly influenced by the Gd_2_O_3_/HfO_2_ heterointerface, rather than random local inhomogeneities. The design of such abrupt, compositionally sharp interfaces is known to be crucial for modulating oxygen vacancy formation energies and migration barriers, which directly impact the non‐volatility and retention performance of memristive devices [33]. The evolution of surface morphology as a function of annealing is elucidated by AFM images shown in Figure 1d,e. Prior to thermal treatment, the oxide film exhibits moderately high but uniform surface roughness, indicative of dense nucleation during initial growth. Post‐annealing at 500°C in argon for 2 h, there is a pronounced reduction in root mean square roughness from 1.76 to 1.70 nm and peak‐to‐valley height from 27.3 to 17.9 nm, as quantified in Table S1. This morphological refinement is attributed to thermally driven grain coalescence and surface energy minimization. From a device physics perspective, smoother surfaces reduce the prevalence of localized field enhancements and random nucleation sites, thereby promoting more uniform formation and rupture of conductive filaments across the device area. This not only improves the uniformity of the SET/RESET voltages but also enhances device endurance and stability, critical parameters for reliable neuromorphic operation [34, 35]. All high‐temperature steps are performed before Ag deposition. To further distinguish the respective roles of annealing and interface engineering, we compare the bilayer device performance before and after thermal treatment, as well as with single‐layer control devices. As shown in Figure S3, the pre‐annealed bilayer device exhibits larger variability in switching voltages and reduced stability, indicating that annealing improves film quality, defect uniformity, and switching consistency. In contrast, single‐layer devices (Figures S4 and S5) show significantly lower ON/OFF ratios and inferior switching characteristics, demonstrating that annealing alone is insufficient to achieve the observed performance. These comparisons indicate that the bilayer Gd_2_O_3_/HfO_2_ interface plays a critical role in enabling spatially confined filament formation and enhanced memory window, while annealing primarily improves material quality and stability.

The XRD pattern for the ALD‐grown HfO_2_ film, as shown in Figure 2a, reveals intense, well‐defined peaks at 2θ = 28.3°, 31.6°, and 54.4°, corresponding to the (‐111), (020), and (311) planes of monoclinic HfO_2_(JCPDS #34‐104). The monoclinic phase of HfO_2_ is further confirmed by Raman spectroscopy: Figure S9a shows bands at ∼252 and ∼302 cm^−^^1^; the ∼302 cm^−^^1^ mode is a well‐known monoclinic‐HfO_2_ feature in thin films [36, 37], while the weaker band near ∼252 cm^−^^1^ has been explicitly reported in monoclinic‐rich HfO_2_ and increases with laser power/ordering [38]. This phase is recognized for its high dielectric constant and thermodynamic stability in memory devices [34, 39, 40]. The sputtered Gd_2_O_3_ layer shown in Figure 2b displays reflections at 2θ = 29.12°, 33.1°, 56.2°, and 65.8°, matching the (222), (400), (046), and (642) planes of the cubic Gd_2_O_3_ (JCPDS #00‐012‐0797). Consistently, Figure S9b exhibits Raman peaks at ∼364 and ∼412 cm^−1^, in agreement with the cubic (bixbyite, *Ia*‐3) fingerprint [41, 42, 43]. Taken together, the sharp, phase‐pure XRD reflections and corroborating Raman signatures with no evidence of secondary phases confirm the structural integrity and clean phase isolation of the individual oxides, providing a robust foundation for the interface‐driven resistive switching [44, 45].

XPS analysis, as shown in Figure 2c‐i and Table S2, further elucidates the oxidation state and defect structure of the constituent layers. The Hf 4f spectrum shown in Figure 2c exhibits doublets at 16.8 eV (Hf 4f_7/2_) and 18.6 eV (Hf 4f_5/2_), confirming that the fully oxidized Hf^4+^ state confirms stoichiometric HfO_2_, while a sub‐peak near 17–18 eV is attributed to non‐stoichiometric HfO_x,_ indicative of an oxygen‐deficient region. This controlled presence of sub‐stoichiometric sites is critical, as these act as potential nucleation points for conductive filaments, underpinning stable resistive switching [46, 47]. The O 1s spectra in both oxides provide a direct measure of the oxygen vacancy population: For HfO_2_, as shown in Figure 2d, a dominant peak at 529.6 eV is assigned to lattice oxygen (O_L_), while a secondary peak at 532.2 eV corresponds to oxygen vacancies (O_v_). A similar trend is observed for Gd_2_O_3,_ shown in Figure 2f, with O_L_ at 529.5 eV and O_v_ at 531.5 eV. The quantitative ratio of these components, derived from peak fitting, serves as a proxy for the defect concentration in each layer, a parameter known to influence the activation energy for ion migration, SET/RESET thresholds, and the retention of conductance states [48, 49]. These data reinforce the view that both HfO_2_ and Gd_2_O_3_ layers possess engineered oxygen vacancy profiles, which are critical for forming and modulating the conductive filaments responsible for resistive switching.

The Gd 4d spectrum, as shown in Figure 2e, presents clear peaks at 145.5 eV (Gd 4d_5/2_) and 141.0 eV (Gd 4d_3/2_), confirming the Gd^3+^ oxidation state and the high chemical purity of the Gd_2_O_3_ film. The XPS survey spectra in Figure 2g,h, show no significant presence of extrinsic contaminants, while the minor C 1s feature at 284.8 eV shown in Figure 2i, is consistent with surface‐adsorbed adventitious carbon, a ubiquitous but inconsequential artifact in oxide film characterization [50, 51]. These comprehensive structural and chemical analyses, supported by the detailed binding energy and assignment data in Table S2, confirm that the bilayer Gd_2_O_3_/HfO_2_ device stack exhibits phase‐pure, compositionally sharp, and defect‐engineered oxide layers. The combined effect of interface‐defined filament confinement and annealing‐induced defect optimization provides a robust platform for achieving reliable and multi‐level resistive switching. In particular, the bilayer interface governs filament dynamics, while annealing enhances structural quality and stability, together enabling the observed device performance [47, 52, 53, 54].

### 2.2 Electrical Characterization and Switching Properties

**FIGURE 3:**
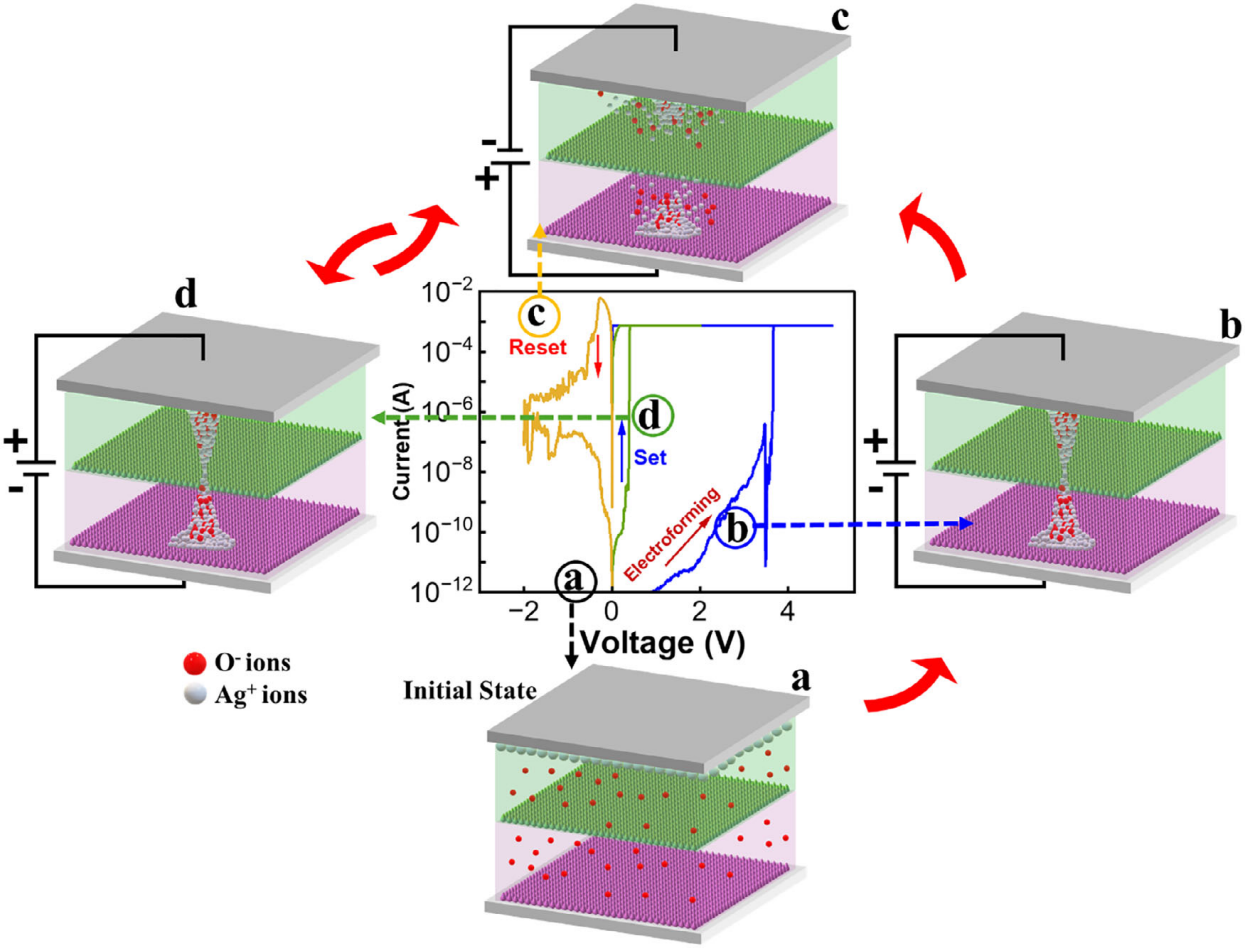
Schematic illustration of the resistive switching cycle in the Ag/Gd_2_O2/HfO_2_/Pt memristor and its correlation with DC *I‐V* characteristics. Central plot representative DC *I‐V* curve of the device, with regions a‐d corresponding to key switching states, each linked to the adjacent schematic. (a) Pristine state (HRS, no bias): Oxygen ions (red) are randomly distributed in the Gd_2_O_3_ top and HfO_2_ bottom layers, and no conductive filament is present. (b) Electroforming (positive bias): A high positive voltage drives Ag^+^ and oxygen ions to form a metallic filament bridging the electrodes, thereby switching the device to LRS. (c) Reset (negative bias): Reverse bias ruptures the filament as oxygen ions migrate back, returning the device to HRS. (d) Set (positive bias): A subsequent positive voltage reforms the filament, enabling repeatable switching between HRS and LRS. Red arrows indicate the cyclic and reversible nature of the switching process.

The resistive switching behavior of the Ag/Gd_2_O_3_/HfO_2_/Pt bilayer memristor is elucidated in Figure 3, which schematically correlates the device's DC I‐V characteristics with the underlying physical and chemical state transitions. These mechanisms are further rationalized using the energy band alignment and process schematics in Figure S1. In the initial pristine state, as shown in Figure 3 (region a), the device exhibits a high‐resistance state (HRS). Here, no continuous filament is present, and oxygen ions are uniformly distributed within the Gd_2_O_3_ and HfO_2_ layers. The energy band diagram in Figure S1a demonstrates clear band bending at both the Gd_2_O_3_/HfO_2_ and oxide/electrode interfaces, as well as a staggered alignment of the conduction and valence bands across the stacks. The Fermi level equilibrates according to the work functions of the electrodes (Ag and Pt), resulting in a built‐in potential profile that governs initial carrier injection and sets the stage for subsequent filament formation. The significant band offsets at the Gd_2_O_3_/HfO_2_ interface act as energy barriers for both electrode and ion transport, localizing electric field enhancement and defect accumulation at the engineered interface. Upon application of a positive bias to the Ag electrode, as shown in Figure 3 (region b) and Figure S1b, Ag atoms oxidize to Ag^+^ and drift through the Gd_2_O_3_ layer under the influence of the local electric field. Oxygen vacancies (O_v_) simultaneously migrate and interact with Ag^+^, promoting the formation of a metallic filament that bridges the electrodes. The resistive switching in this device is primarily governed by Ag filament formation, consistent with an electrochemical metallization (ECM) mechanism. Oxygen vacancies play a secondary role by modulating the local electric field and assisting filament nucleation, particularly at the Gd_2_O_3_/HfO_2_ interface. The sharp Gd_2_O_3_/HfO_2_ interface modulates vacancy formation energy and provides a preferred site for initial filament nucleation; this is a direct result of the band offset and local defect chemistry at the boundary. This transition from HRS to (LRS) is associated with a sudden increase in current during the SET process. Switching from LRS back to HRS, as shown in Figure 3 (region c) and Figure S1c, is induced by reversing the bias. During the RESET process, the metallic filament is ruptured primarily due to the reverse migration of Ag^+^ ions toward the top electrode, while oxygen vacancy redistribution assists in restoring the high‐resistance state. The dynamic evolution of band bending during this RESET process alters the energy barrier landscape, facilitating controlled dissolution of the filament and returning the device to its initial state. The reversible nature of these processes is illustrated by the cyclic sequence of switching events in Figure 3. The conduction characteristics in each resistive state can be rigorously described using transport models for oxide‐based memristors. In the LRS, after filament formation, charge transport is dominated by metallic conduction along the filament path, yielding a linear relationship between current and voltage. This behavior is consistent with conduction through a metallic Ag filament bridging the electrodes.

$$I_{LRS}=k_{ohm}V$$

where *k_ohm_*, is the conductance of the metallic filament. This ohmic regime is confirmed by a log‐log slope of ∼1 in experimental I‐V data, reflecting direct, low‐barrier carrier transport between electrodes [46, 47, 55, 56]. In contrast, the HRS, prior to filament formation or following filament rupture, is governed by space‐charge‐limited current (SCLC) transport. In this regime, the injected carrier density surpasses the intrinsic carrier density, and charge is progressively trapped and detrapped within the oxide layer, particularly at sites associated with oxygen vacancies. The current thus follows a power law with respect to voltage:

$$I_{HRS}=k_{SCLC}V^{n}$$

where *k_SCLC_* is a pre‐factor dependent on material properties and trap density, and the exponent *n*, is indicative of the trap distribution. For a uniform trap distribution, *n*  =  2. However, in the presence of an exponential distribution of deep traps or strong disorder, *n* may increase further, often reaching values of 3 to 4, as observed in our devices [46, 47]. This behavior is consistent with trap‐filled SCLC theory, and the value of *n* can be related to the characteristic temperature of the trap distribution via *n*  =  1 +  *T_c_*/*T*, where *T*is the absolute measurement temperature, and *T_c_* is the characteristic temperature associated with the trap energy distribution in the oxide [47]. The SCLC regime is therefore highly sensitive to defect chemistry and interface engineering in the oxide stack. This indicates that, in the absence of a continuous metallic filament, charge transport is governed by trap‐controlled conduction associated with oxygen vacancies within the oxide layers.

Crucially, the engineered band offsets, Fermi level alignment, and associated band bending at the Gd_2_O_3_/HfO_2_ interface are instrumental in spatially confining filament formation, regulating the local electric field, and enhancing the reproducibility of resistive switching. The atomically sharp Gd_2_O_3_/HfO_2_ interface further enhances this switching behavior by modulating local electric fields, acting as an energetic barrier for both oxygen vacancy and metal ion migration. This interface engineering results in spatial confinement of filament formation, improved reproducibility of SET/RESET transitions, and a pronounced reduction in device‐to‐device and cycle‐to‐cycle variability. The role of such an engineered interface in suppressing undesired filament overgrowth and enabling multi‐level conductance tuning is now well established for neuromorphic and memory device applications [52, 57]. Thus, the resistive switching mechanism is dominated by Ag filament formation, while oxygen vacancies regulate filament nucleation, confinement, and rupture dynamics at the engineered interface.

**FIGURE 4:**
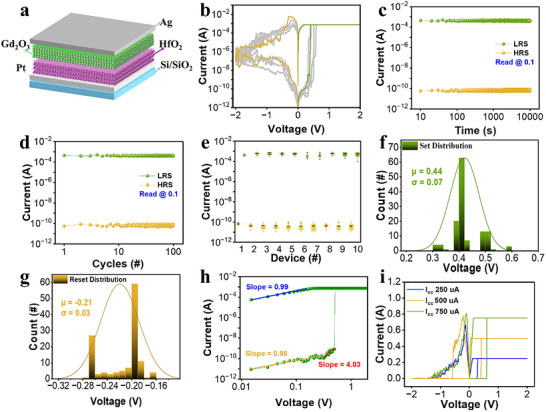
Electrical performance of the Ag/Gd_2_O_3_/HfO_2_/Pt memristor device. (a) Schematic illustration of the device architecture, showing the Ag top electrode, Gd_2_O_3_, and HfO_2_ functional layers, and Pt bottom electrode. (b) Typical DC I‐V characteristics over multiple cycles illustrating stable bipolar switching, where colored traces highlight the first set and reset cycles after electroforming. (c) Long‐term retention characteristics of the high‐resistance (HRS) and low‐resistance (LRS) states at a read voltage of 0.1 V, measured over 10,000 s. (d) Endurance performance over 100 consecutive switching cycles at 0.1 V, exhibiting highly stable and distinctly separated HRS and LRS. (e) Cycle‐to‐cycle variability of HRS and LRS read currents measured across 10 individual devices (100 cycles per device), at a read voltage of 0.1 V, confirming excellent device‐to‐device uniformity and reproducible switching behavior. (f) Statistical distribution of SET voltages, fitted with a Gaussian profile (µ = 0.44 V, σ = 0.07), evidencing tight switching voltage control and low cycle‐to‐cycle variability. (g) Gaussian‐fitted distribution of RESET voltages (µ = ‐0.21 V, σ = 0.03), further confirming narrow switching parameters dispersion and high operational uniformity. (h) Log‐log plot of I‐V characteristics, demonstrating ohmic conduction behavior (slope ≈ 0.99) and space‐charge‐limited conduction (SCLC) with (slope ≈ 4.03), consistent with filamentary switching mechanisms. (i) Compliance current‐dependent switching behavior (250µA, 500µA, and 750µA), demonstrating the devices multilevel resistive switching capability.

The robust resistive switching and electrical reliability of the Ag/Gd_2_O_3_/HfO_2_/Pt bilayer memristor are comprehensively demonstrated in Figure 4, with further insights provided in Figures S2–S5. The device architecture, shown schematically in Figure 4a, features vertically stacked Ag, Gd_2_O_3_, HfO_2_, and Pt layers. This carefully engineered configuration, as detailed in the structural analysis, shown in Figure 1, ensures spatial separation of the functional oxides while localizing electric field concentration and defect accumulation at the Gd_2_O_3_/HfO_2_ interface. This interface serves as a critical site for regulating filament nucleation and growth, a key factor in achieving resistive switching. Figure 4b presents representative DC *I‐V* curves, revealing stable, repeatable bipolar switching cycles with tightly clustered SET and RESET voltages. This reliable switching reflects the combined effect of annealing‐induced defect optimization and interface‐engineered filament confinement, where annealing improves material uniformity and stability. At the same time, the atomically sharp interface governs filament localization and switching dynamics. In contrast, Figure S3, corresponding to the same device structure before annealing, shows greater variability in switching voltages, less distinct SET/RESET transitions, and higher cycle‐to‐cycle fluctuation. The enhanced performance after annealing can be attributed to grain coalescence, improved crystallinity, and reduced surface roughness, all of which sharpen the oxide‐oxide interface and suppress the stochastic nature of filament growth. Importantly, all high‐temperature steps were completed before Ag deposition, and no post‐metal heating was applied; this limits thermal exposure of peripheral circuitry and reduces the risk of lateral Ag diffusion under the operating conditions used here. The physics underlying this improvement is rooted in the fact that a smooth, well‐defined interface minimizes random local electric field enhancements, confines oxygen vacancy migration, and thereby increases switching uniformity.

Non‐volatile retention of both HRS and LRS over 10,000 s at a 0.1 V read voltage is shown in Figure 4c. The near‐constant current levels highlight excellent data retention, as expected from the filament‐confined interface‐stabilized switching mechanism, and minimal drift from environmental or intrinsic charge trapping effects. Figure 4d and Figure S2 detail the endurance and statistical uniformity of the resistive switching characteristics. Over 100 consecutive switching cycles, the device maintains highly stable and well‐separated HRS and LRS current states, indicative of excellent endurance. Figure S2a shows the cumulative probability distributions of HRS and LRS read currents for a single device, measured at a read voltage of 0.1 V, demonstrating a clear separation with an ON/OFF ratio approaching 10^7^. The corresponding cycle‐to‐cycle variability is analyzed in Figure S2b box plots, which yield a coefficient of variation (CoV) of 0.204 for HRS and 0.0645 for LRS, reflecting reduced dispersion and high switching consistency within a single device. To further assess device‐to‐device statistical reliability, Figure 4e shows box plots of HRS and LRS read currents obtained from 10 separate devices, each measured over 100 cycles. The calculated CoV among the 10 devices is 0.23 for HRS and 0.12 for LRS, indicating low inter‐device variability. Additionally, all devices exhibit a stable ON/OFF current ratio approaching 10^7^, further confirming excellent reproducibility, uniform resistive switching behavior, and robust fabrication control. Figure S3 (pre‐annealing) shows a reduced ON/OFF current ratio (10^3^–10^5^) and large variability. The ON/OFF ratio after annealing, with minimal interquartile range fluctuation, confirms the device's suitability for high‐performance non‐volatile and neuromorphic applications. Figures S4 and S5 (single‐layer HfO_2_ and Gd_2_O_3_ controls) show significantly lower ON/OFF ratios (10^2^–10^3^), underscoring the necessity of bilayer interface engineering for optimal device performance. Gaussian‐fitted set and reset voltage distribution as shown in Figure 4f,g, reflecting a tight cycle‐to‐cycle variability and statistical reliability (µ_Set_ = 0.44 V, σ_Set_ = 0.07; µ_Reset_ = ‐0.21 V, σ_Reset_ = 0.03). In contrast, Figure S3d,e, the pre‐annealed device shows broader distributions (µ_Set_ = 1.25 V, σ_Set_ = 0.80; µ_Reset_ = ‐0.80 V, σ_Reset_ = 0.60), highlighting the stabilizing effect of both annealing and bilayer structure. The log‐log I‐V plot in Figure 4h shows that LRS transport follows ohmic conduction (slope ≈ 0.99). confirming metallic filamentary behavior, while HRS transport follows SCLC (slope ≈ 4.03), indicating trap‐controlled current in the oxide. This transition was modeled quantitatively in the previous section, directly linked to the defect landscape and interface‐modulated energy barriers revealed in the structural and XPS analysis. This dual‐regime conduction is essential for enabling both digital (binary) and analog (multi‐level) memory states, as required for neuromorphic hardware. The multilevel switching capability of the bilayer memristor is demonstrated in Figure 4i, where varying the compliance current during the SET process (250 µA, 500 µA, and 700 µA) enables controlled modulation of the device's low resistance state. This multilevel control also enables array‐level mitigation of sneak currents. Lowering the SET compliance increases the LRS resistance, thereby reducing selected‐cell read current and suppressing half‐select leakage paths. At a low read voltage of 0.1 V, the device maintains a large read margin (ON/OFF ≈ 10^7^) over 100 cycles, with minimal intra‐ and inter‐device variability supporting reliable low‐bias sensing. These device‐level attributes align with established crossbar strategies such as 1T1R/1S1R architectures, self‐rectifying designs, V/2–V/3 biasing, and write‐/read‐verify protocols for managing sneak paths. Together, the robust read margin, tunable resistance states, and statistical uniformity form a strong device‐level foundation for future crossbar integration.

### 2.3 Integration with Neuromorphic and AI Simulation

**FIGURE 5:**
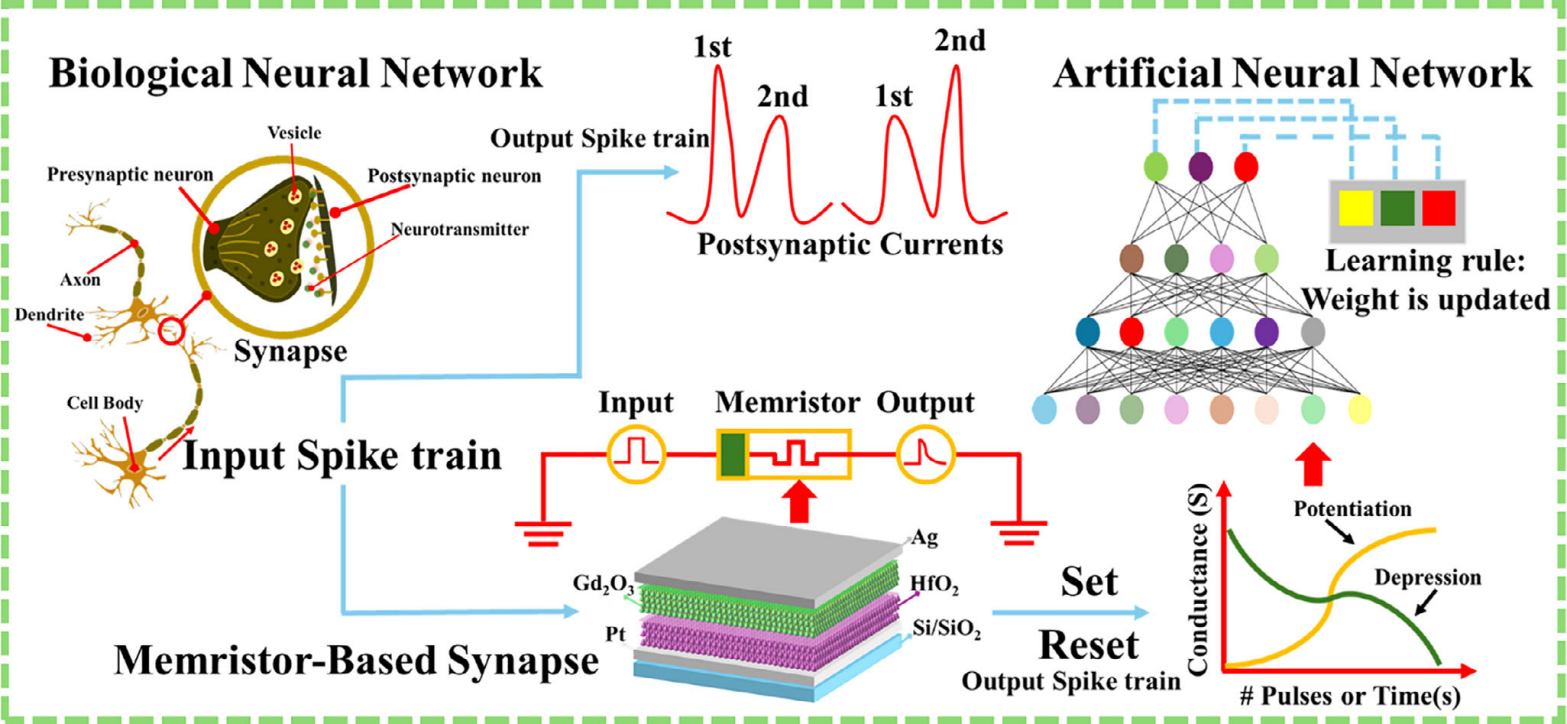
Schematic of neuromorphic information processing enabled by memristor‐based artificial synapses, inspired by biological synapses. A biological neural network transmits input spike trains that, upon reaching the synapse, induce changes in post‐synaptic current, thereby modulating synaptic strength through potentiation or depression. Analogously, electrical spike inputs applied to the memristor device result in conductance changes (potentiation or depression) that emulate synaptic weight updates. These adjustable conductance states are used as trainable weights in artificial neural networks, enabling cognitive tasks such as digit, pattern, image, and color recognition, with weight‐update rules inspired by biological synaptic plasticity.

Modern AI and sensory applications increasingly require hardware platforms that can efficiently perform parallel, event‐driven computation, mimicking adaptive and plastic information processing in biological neural networks. At the core of such networks, the biological synapse operates as a dynamic analog connection, modulating its weight (efficacy) in response to the timing and frequency of action potentials, thereby enabling learning and memory formation. Figure 5 schematically illustrates this conceptual bridge between biology and neuromorphic hardware. In the left panel, the synapse lies at the heart of biological intelligence, where action potentials (spikes) from a pre‐synaptic neuron drive the release of neurotransmitters, resulting in a change in post‐synaptic current. This change, whether potentiation or depression, encodes a plastic weight update, which forms the physical basis for learning and adaptation in neural systems.

The bottom panel of Figure 5 depicts the artificial analog: an Ag/Gd_2_O_3_/HfO_2_/Pt memristor‐based synapse. Here, electrical spike pulses, functionally analogous to biological action potentials, are applied to a memristor device. These pulses induce gradual, controlled changes in the device conductance, directly emulating the synaptic plasticity seen in biology. Thus, the memristor conductance functions as an analog, programmable synaptic weight, and conductance modulation captures both potentiation and depression. The flow of information in this neuromorphic framework is represented visually in Figure 5, where input spike trains are transduced into conductance changes in the memristor synapse, and these conductance states are subsequently mapped as weights in an artificial neural network. This direct mapping enables hardware‐level learning and inference, laying the foundation for efficient, low‐power implementations of AI tasks such as digit, pattern, and color recognition.

**FIGURE 6:**
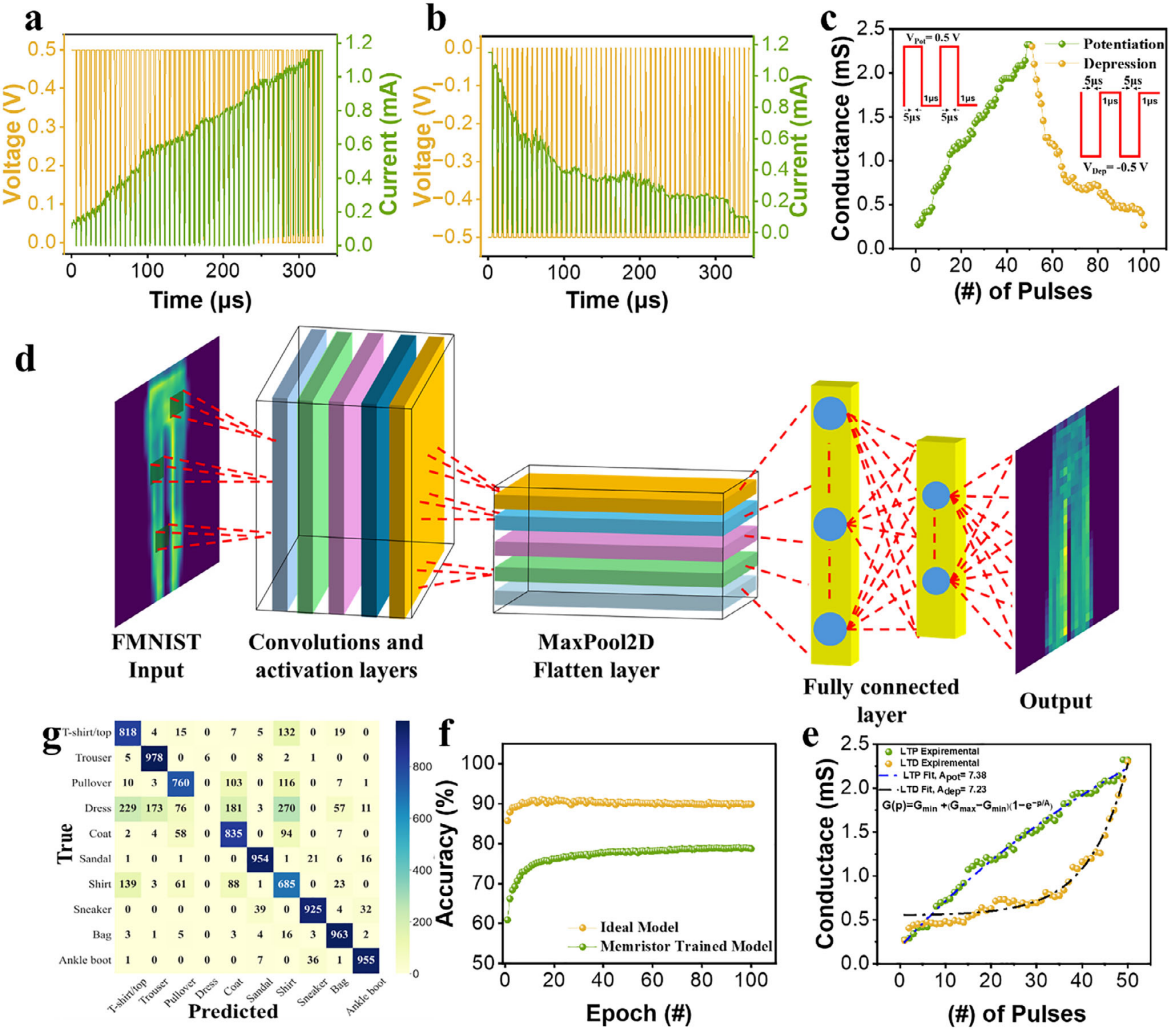
Synaptic plasticity and hybrid memristor‐CNN simulation results for Fashion‐MNIST classification. (a) Device current response (green) under consecutive positive voltage pulses (0.5 V, 5 µs width, 1 µs delay), showing a gradual conductance increase (potentiation) over 50 pulses. (b) Device current response (green) under negative voltage pulses with identical parameters, exhibiting a progressive conductance decrease (depression) over 50 pulses. (c) Conductance changes during potentiation and depression, with the inset illustrating the pulse protocols used for each process. (d) Schematic of the hybrid CNN architecture integrating a memristor‐inspired classifier layer; input Fashion‐MNIST images are processed through convolution layers, and the final weights are updated based on the experimentally fitted device response. (g) Test accuracy (78%) versus training epoch curves comparing the hybrid memristor‐CNN with an ideal (89%) digital CNN baseline, illustrating robust learning and the trade‐off between analog device realism and classification precision. (e) Exponential fit of experimental conductance data for potentiation (A_pot_ = 7.38) and depression (A_dep_ = 7.23), validating the physical memristor model used in network simulation.

**FIGURE 7:**
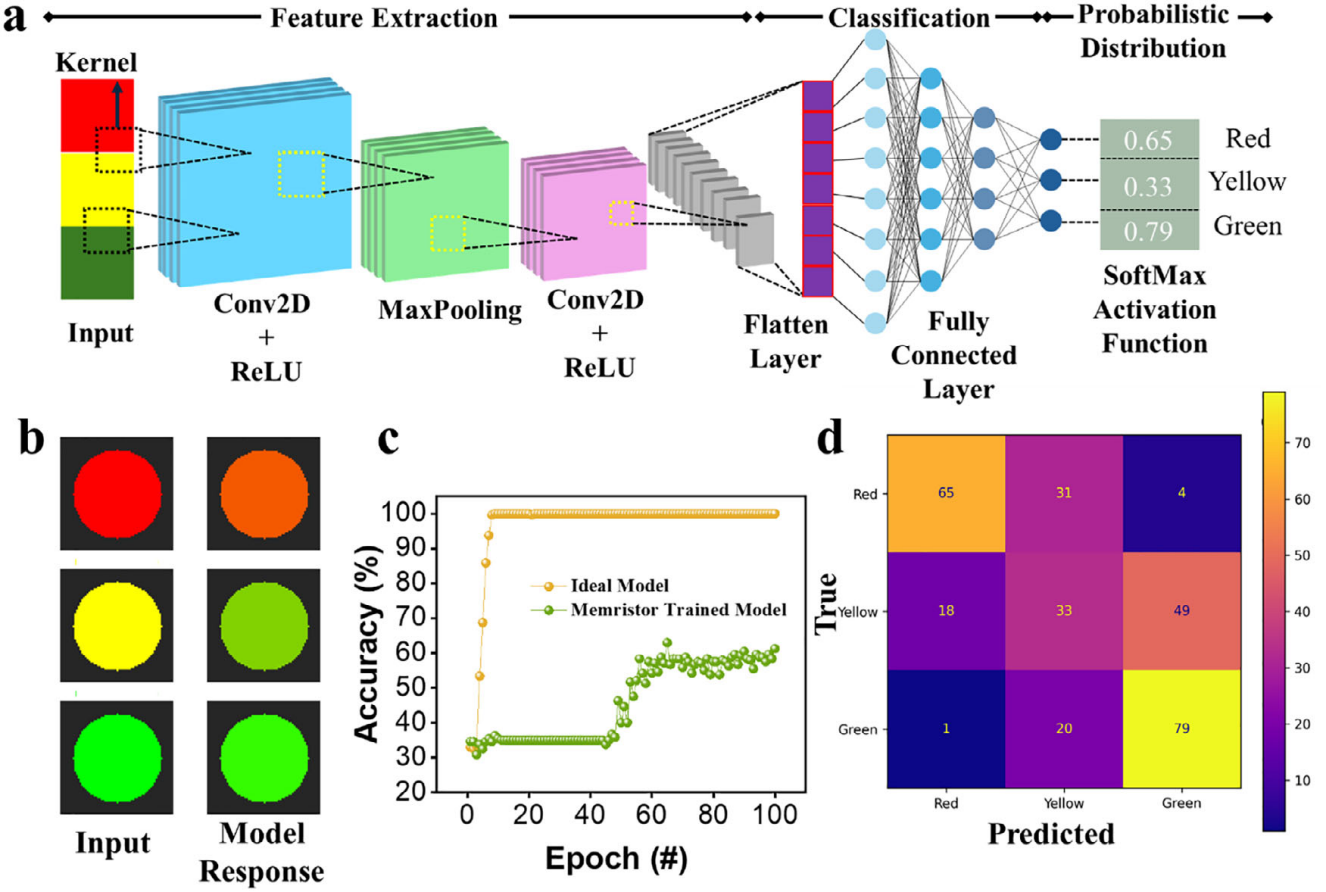
Hardware‐constrained color recognition using memristor‐based convolutional neural network (CNN) models. (a) Schematic of the CNN architecture used for color pattern classification. Input 7 × 7 pixel conductance maps representing traffic signal colors (red, yellow, green) are mapped to output classes through two conventional layers, a max‐pooling layer, a flatten layer, and two layers, and output softmax layers, yielding the final class probabilities (0.65 red, 0.33 yellow, and 0.79 green). (b) Input color patterns and model output responses, illustrating the classification pipeline for both ideal and memristor‐derived conductance data. (c) Test accuracy versus training epoch for both ideal (noise‐free) and memristor‐based CNN models. (d) Confusion matrix for the memristor‐based CNN, showing class‐specific accuracy: 65% for red, 33% for yellow, and 79% for green, with most misclassifications between yellow and green due to overlapping conductance profiles.

To provide a rigorous link between synaptic device physics and network learning, both our F‐MNIST (Figure 6) and color recognition (Figure 7) simulations employ a unified pulse‐driven exponential model. In both biological and artificial systems, synaptic efficacy (weight) can be described by an exponential dependence on spike activity or pulse count. For our memristor, the conductance evolution with pulse number is modeled by:

$$G\left(p\right)=G_{\min}\;+\left(G_{\max}-\;G_{\min}\right)\left[1-\;e^{-P/A}\right]$$

where *G_min_* and *G_max_* represent the minimum and maximum conductance states of the device, *p* is the number of applied potentiation (or depression) pulses, and *A*is a characteristic parameter (distinct for potentiation and depression and extracted directly from device measurements). This equation is used throughout our neuromorphic simulations, enabling direct translation of experimental device behavior into neural network weights, ensuring that learning and inference authentically reflect the underlying memristor physics. By anchoring both F‐MNIST and color recognition simulation to this unified model, we established a robust quantitative bridge between spike‐driven plasticity in the memristor and real‐world cognitive computation in artificial neural networks. This not only validates the conceptual analogy illustrated in Figure 5 but also demonstrates its practical scalability and versatility for diverse AI tasks showcased in the subsequent sections.

The synaptic plasticity characteristics of the Ag/Gd_2_O_3_/HfO_2_/Pt memristor were directly leveraged in a hardware‐constrained convolutional neural network (CNN) for the Fashion‐MNIST classification task, as shown in Figure 6. Long‐term potentiation (LTP) and depression (LTD) characteristics, as shown in Figures 6a,b, were acquired via single‐shot measurements using +0.5 V pulses for potentiation and −0.5 V pulses for depression (5 µs in width, 1 µs interval, 50 pulses per cycle). The dynamic evolution of device conductance is shown in Figure 6c under alternating potentiation and depression pulse trains over 50 cycles each, with the inset schematic visualizing the pulse for both processes. The exponential pulse‐driven model accurately captured the experimental conductance changes, as shown in Figure 6e, with device‐specific parameters (*A_pot_* =  7.38,   *A_dep_* =  7.23) and endpoints *G_min_* and *G_max_* representing the measured conductance extremes. The resulting conductance states were mapped directly without normalizing or rescaling into classifier weights of a CNN comprising two convolutional layers (32 and 64 filters, 3 × 3 kernels, ReLU activation), a max pooling layer, a flattening layer (1600 features), and a memristor‐based classifier as schematically shown in Figure 6d. During network training, the convolution layers were updated using standard backpropagation, while the classifier layer weights were modulated through a hardware‐inspired, pulse‐based protocol: after each batch, weight increments were log‐scaled and implemented as potentiation or depression pulses, with the resulting conductance determined by the measured LTP/LTD device curves. Performance evaluation on the full F‐MNIST dataset (60,000 training, 10,000 test samples) shows that the memristor‐integrated classifier achieves a peak accuracy of ∼78%, compared to ideal, noiseless models ∼89% as shown in Figure 6f. The difference in performance compared to the ideal model (∼89%) arises from the weight‐mapping scheme employed in this work. In the present implementation, the synaptic weights are derived directly from experimentally measured conductance values, and weight updates are implemented through pulse‐driven modulation based on the measured LTP/LTD characteristics of the device. Additional processing steps, such as normalization, linearization, or compensation, are not incorporated in the mapping procedure. Under these conditions, the network performance is influenced by the characteristics of the device response during training. In particular, nonlinear conductance evolution, asymmetry between potentiation and depression, and the finite number of accessible conductance states affect the effective precision of weight updates, which is reflected in the obtained classification accuracy. A more detailed examination of the device‐driven learning behavior can be obtained from the conductance evolution characteristics in Figure 6e. The exponential LTP/LTD response indicates that conductance updates are inherently nonlinear and asymmetric, resulting in non‐uniform weight update steps during training. As a consequence, identical weight updates in the learning algorithm are translated into unequal conductance changes, effectively introducing an intrinsic update noise during training, which perturbs the gradient‐driven optimization process and limits the achievable weight precision. From a system‐level perspective, this behavior manifests as an effective noise source during training, as reflected in Figure S7. The non‐uniform ΔG distribution indicates spatial variability in weight updates across synapses, while the broadened output probability distribution reflects reduced confidence and stability in network predictions. This correlation provides a direct link between device‐level nonlinearity and its impact on learning dynamics and classification performance. Confusion matrix analysis, as shown in Figure 6g, highlights class‐dependent trends, such as higher error rates in classes with visually similar patterns and higher sensitivity to conductance quantization, while the temporal accuracy plot shows convergence stability across epochs. To further probe how memristor‐induced analog constraints affect neuromorphic learning, Figure S7 provides two complementary analyses. Figure S7a visualizes the average weight‐update map (ΔG) for all input‐output connections in the classifier, highlighting the distribution and adaptation of synaptic weights during network training. Figure S7b presents the output class probability vector for a representative test sample, illustrating how hardware‐driven variability influences classification confidence and uncertainty in model predictions. Together, these visualizations reveal that the non‐idealities of real memristive hardware propagate through the network, shaping both the internal feature learning and the reliability of output decisions. To further contextualize the obtained accuracy, a comparison with recently reported memristor‐based neuromorphic systems is summarized in Table S3, highlighting the influence of weight‐mapping strategies on classification performance.

To further evaluate the practical neuromorphic capability of the Ag/Gd_2_O_3_/HfO_2_/Pt memristor, we implemented a hardware‐driven color recognition system using direct device measurements as synaptic weights in a compact convolutional neural network (CNN), as schematically illustrated in Figure 7a. The task simulates traffic signal detection, with red, yellow, and green stimuli on a 7 × 7 pixel grid, each color represented by a distinct set of potentiation pulses mapped to experimentally extracted conductance values (red:10, yellow:17, green: 23 pulses), ensuring that all the coding remains within the measured device LTP window. The full workflow, as shown in Figure S8a, maps the conductance states of the memristor acquired via a single‐shot LTP protocol (+0.5 V, 5 µs in width, 1 µs interval, 50 pulses) directly to the CNN's input layer, with no rescaling or normalization, preserving physical device extremes and realistic analog variability. As shown in Figure 7b, representative input stimuli and their real model responses illustrate the system's end‐to‐end inference, with green, yellow, and red inputs producing distinct output distributions, but with observable uncertainty and blending in the output for ambiguous cases. The CNN architecture, as shown in Figure 7a consists of two convolutional layers (16 and 32 filters, 3 × 3 kernels, ReLU), a max‐pooling layer, flattening, and dense layers (64 units + 3‐class softmax output). Two variants were benchmarked: an ideal model (synthetic, noiseless conductance maps) and a memristor‐driven model (using real device data). The ideal classifier achieves perfect performance (100% accuracy), while the memristor‐based model achieves a maximum validation accuracy of 59%, as shown in Figure 7c. Class‐wise confusion matrix analysis shown in Figure 7d reveals that green samples are recognized most reliably (79% correct), while yellow is most susceptible to misclassification, often confused with green (49%) or red (18%). Red achieves 65% accuracy, with the remaining errors distributed primarily to yellow. These trends reflect the non‐linear and asymmetric analog conductance response of the memristor to pulse number, and the partial overlap in the physical LTP conductance curves for the yellow and green input pulse regions. To further probe network performance, Figure S8b shows a synaptic weight change heatmap for the Dense (64) layer, revealing distributed and adaptive weight adjustment in response to device noise and input ambiguity. Despite analog constraints, the network learns to reweigh input channels to maximize separability.

Benchmarking underscores the competitive advantage of our interface‐engineered structure in meeting the critical performance metrics required for next‐generation non‐volatile and neuromorphic memory technologies. Table 1 provides a comparison of the memristor device with recently reported oxide‐based bilayer devices, highlighting its superior ON/OFF ratio, fast switching, low programming energy, and demonstrated neuromorphic performance.

**TABLE 1:** Comparison of key performance parameters for our device with recently reported oxide‐based bilayer memristors.

Device Structure	ON/OFF Ratio	Retention (s)	Switching Time	Energy/event (pJ)	Set/Reset Voltage (V)	Multilevel/Simulation Demonstrated	Refs.
Gd_2_O_3_/HfO_2_	10^7^	> 10^4^	350 ns	13.6	0.44 / −0.21	LTP/LTD, F‐MNIST & color recognition simulations	**This work**
HfO_2_/Ta_2_O_5_	∼104	≥ 10^4^	—	—	0.92 / −1.10	LTP/LTD analog, STDP	[58]
TaOx/TiOy/Ti	∼6.6 × 10^2^	—	—	12,690	1.2 / −1.3	Digit recognition	[59]
TiO_x_N_y_/SnO_x_	∼10^2^	> 10^3^	∼ 400 ns	3,240 pJ	1.5/ −1.3	Digit recognition	[60]
TiN/IGZO/ZnO	∼5	10^4^	—	—	0.55 / −0.47	—	[61]
NiO/Nb_2_O_5‐x_	∼ 10^4^	>10^4^	—	—	1.7 /−1.2	—	[62]
TiO_x_/BaTiO_3_	∼50	>10^4^	—	1.76 pJ	0.6/−1.2	Digit recognition	[63]
AuNp‐doped DNA/HfO_2_	∼10^4^	10^4^	—	—	1.5/−2.2	Digit recognition	[64]
GaSe/HfO_2_	∼10^5^	—	—	—	3/−1.5	Digit recognition	[65]
β‐Ga_2_O_3_/WO_3_	∼182	10^3^	—	—	+0.67/−0.35	—	[66]
Ag(Ag+)‐V_2_O_5_/SnO_2_	10^4^	10^5^	—	1.67 fJ	1.96/−1.76	Image recognition	[67]

## 3 Conclusion

Our study establishes a new paradigm in oxide‐based memristive technology by uniting materials interface engineering with experimentally validated neuromorphic system performance. By leveraging the unique dielectric, defect, and interface properties of rare‐earth (Gd_2_O_3_) and high‐κ (HfO_2_) oxides, our device exhibits non‐volatile memory performance with an ultra‐high ON/OFF current ratio (∼10^7^), reliable state retention (>10^4^ s), a rapid switching transition time (350 ns), and low programming energy (13.6 pJ). The engineered Gd_2_O_3_/HfO_2_ interface not only suppresses random filament formation but also stabilizes multilevel conductance states essential for synaptic plasticity and analog learning.

Comprehensive structural and electrical characterization reveals atomically abrupt interfaces, reduced roughness, and precisely modulated defect landscapes, enabling highly uniform SET/RESET switching and cycle‐to‐cycle repeatability. Statistical analysis confirms tight switching parameter distributions, minimal device variability, and large memory windows that far exceed those of single‐layer oxide controls and previously reported bilayer devices. Quantitative modeling and supplementary pulse‐driven experiments further elucidate the underlying physics, including interface‐modulated band offsets, filament confinement, and space‐charge‐limited conduction in the high‐resistance state. Most notably, we directly mapped measured device plasticity into artificial neural network simulations, achieving hardware‐constrained Fashion‐MNIST (F‐MNIST) classification with a peak accuracy of 78% and demonstrating robust color recognition in noisy analog regimes. The memristor's analog nonlinearity and weight update dynamics are shown to shape the learning trajectory, feature representation, and uncertainty in model predictions, underscoring the critical importance of physical device constraints for system‐level neuromorphic performance.

Overall, our results establish that rare‐earth/high‐κ oxide bilayer memristors with engineered interfaces, fast and energy‐efficient switching, and experimentally validated synaptic plasticity offer a scalable and technologically viable platform for next‐generation non‐volatile memory and edge‐AI hardware. Building on this foundation, future efforts will focus on further enhancing device reliability and system‐level performance through continued interface optimization and improved integration with neuromorphic architectures. In particular, extending this approach toward large‐scale crossbar arrays and hardware‐aware learning frameworks represents a promising direction for translating device‐level functionality into practical, high‐performance neuromorphic systems.

## 4 Experimental Section

### 4.1 Device Fabrication

Bilayer memristors with the structure Ag/Gd_2_O_3_/HfO_2_/Pt were fabricated on commercially available Pt‐coated Si/SiO_2_ substrates. Substrates were sequentially cleaned by ultrasonication in acetone, isopropanol, and deionized water for 10 min each, followed by N_2_ drying and a 120°C bake to remove surface moisture. The 28 nm HfO_2_ switching layer was grown by thermal ALD at 250°C using TEMAHf and H_2_O (oxidant) with Ar as carrier/purge under low‐pressure conditions. Wafers were preheated in‐situ for 600 s at 250 °C (Ar), and each cycle comprised TEMAHf pulse 0.3 s, Ar purge 8 s, H_2_O pulse 0.1 s, Ar purge 8 s, yielding ∼1.0 Å cycle^−1^ for ∼280 cycles to obtain 28 nm. Subsequently, 38 nm Gd_2_O_3_ was deposited by RF magnetron sputtering at 500°C and 140 W from a Gd_2_O_3_ ceramic target. The chamber base pressure was 2 × 10^−6^ Torr, and the working pressure was 2.7 × 10^−2^ (27 mTorr) with Ar/O_2_ = 18/2 sccm. Substrate rotation was enabled, the target was pre‐sputtered for 30 min, and a 60 min run produced a 38 nm film (≈ 0.63 nm min^−1^). The bilayer was then annealed at 500°C for 2 h in Ar. The tube was evacuated and Ar back‐filled three times, annealed under a continuous Ar purge at near‐atmospheric pressure, the furnace ramped to 500 °C ≈ 2°C min^−1^, and cooled naturally to room temperature. All high‐temperature steps preceded Ag deposition, and no post‐metallization thermal processing was performed. The Ag top electrode 200 nm was deposited by thermal evaporation at 2 × 10^−6^ Torr with a ∼2 Å s^−1^ rate through a shadow mask defining 100 × 100 µm^2^ pads; no adhesion/seed layer was used. Single‐layer HfO_2_ and Gd_2_O_3_ control devices were fabricated on the same substrates using the corresponding single‐layer versions of the above processes.

### 4.2 Device Characterization

The structural and morphological properties of the bilayer memristor devices were examined using high‐resolution transmission electron microscopy (HR‐TEM) to assess layer thickness and interface sharpness. Energy‐dispersive x‐ray spectroscopy (EDS) was employed to confirm the elemental distribution across the device stack. Atomic force microscopy (AFM) was used to analyze surface morphology and roughness before and after annealing. X‐ray diffraction (XRD) provided information on crystalline phases, and X‐ray photoelectron spectroscopy (XPS) was used to determine the chemical composition and oxidation states of the constituent layers.

### 4.3 Electrical Measurements

All electrical characterization was carried out at room temperature in ambient air using a Keysight B1500A semiconductor parameter analyzer in conjunction with a probe station, with the Ag top electrode biased and the Pt bottom electrode grounded. Devices underwent a single electroforming step using a quasi‐static voltage sweep up to 5 V with Ag positive; the typical forming voltage was V_form_ ≈ 3.5V _with_ current compliance of I_cc_ = 750 µA applied to prevent hard breakdown. After forming, devices were bipolarly cycled by sweeping from +2 V to −2 V for 100 consecutive DC cycles (SET I_cc_ = 750 µA). Retention and endurance tests were evaluated at a read bias of 0.1 V for up to 10^4^ s and 100 cycles, respectively. Pulse‐driven potentiation and depression were measured using a waveform generator/fast measurement unit (WGFMU), applying voltage pulses of 0.5 V (potentiation) or −0.5 V (depression), 5 µs width, with 1 µs intervals; the device current was recorded in real time.

### 4.4 Neural Network Simulation

Neuromorphic performance was evaluated by directly integrating experimentally measured device plasticity into artificial neural network simulations. For the F‐MNIST and color recognition tasks, custom convolutional neural network (CNN) architectures were implemented in Python using PyTorch. Synaptic weights corresponding to the classifier layer were updated according to the experimentally extracted LTP/LTD curves, using the exponential pulse‐driven model detailed in the main text. During training, conventional backpropagation was used for the convolutional layers, while classifier weights were modulated via pulse translation matching the device conductance change. All experiments used the full F‐MNIST dataset (60,000 training and 10,000 test samples) or custom color‐mapped patterns, with accuracy, confusion matrices, and weight evolution tracked over 100 training epochs.

## Funding

This work was supported by the National Research Foundation (NRF) of Korea, funded by the Ministry of Science and ICT (RS‐2025‐02303505, RS‐2024‐00468995).

## Conflicts of Interest

The authors declare no conflicts of interest.

## Supporting information




**Supporting File**: smll73836‐sup‐0001‐SuppMat.docx.

## Data Availability

The data that support the findings of this study are available in the supplementary material of this article.
