# Supplementary material for: A Bilayer Rare‐Earth/High‐κ Oxide Memristor for Energy‐Efficient Neuromorphic Intelligence
Source: Small. 2026 May 20;22(38):e73836. doi: 10.1002/smll.73836 (PMC13351500; doi:10.1002/smll.73836)
Supplement: Supplementary file 1 — Supporting File: smll73836‐sup‐0001‐SuppMat.docx. [file SMLL-22-e73836-s001.docx]

*Supporting Information*

**A Bilayer Rare-Earth/High-κ Oxide Memristor for Energy-Efficient Neuromorphic Intelligence**

*Hammad Ghazanfar^1,2, #^ , Muhammad Rabeel^2,#^, Honggyun Kim^1,#^ , Sobia Nisar^2^, Muhammad Wajid Zulfiqar^,2,3^Muneeb Ahmad^1,2^*, *Rana Faryad Ali^4^, Ghulam Dastgeer*^5^, Deok-kee Kim*^1,2^*

*^1^Department of Semiconductor Systems Engineering, Sejong University, Seoul, 05006, Republic of Korea.*

*^2^Department of Electrical Engineering and Convergence Engineering for Intelligent Drone, Sejong University, Seoul 05006, Republic of Korea.*

*^3^Graduate School of Optical Engineering, Sejong University, Seoul 05006, Republic of Korea.*

*^4^Department of Materials Science and Engineering and Materials Research Laboratory*

*Massachusetts Institute of Technology (MIT), USA.*

*^5^Department of Physics and Astronomy, Sejong University, Seoul 05006, Republic of Korea.*

*#These authors contributed equally.*

***Corresponding authors****: Ghulam Dastgeer and Deok-kee Kim*

***Email:*** [*gdastgeer@sejong.ac.kr*](mailto:gdastgeer@sejong.ac.kr) *&* [*deokkeekim@sejong.ac.kr*](mailto:deokkeekim@sejong.ac.kr)

**
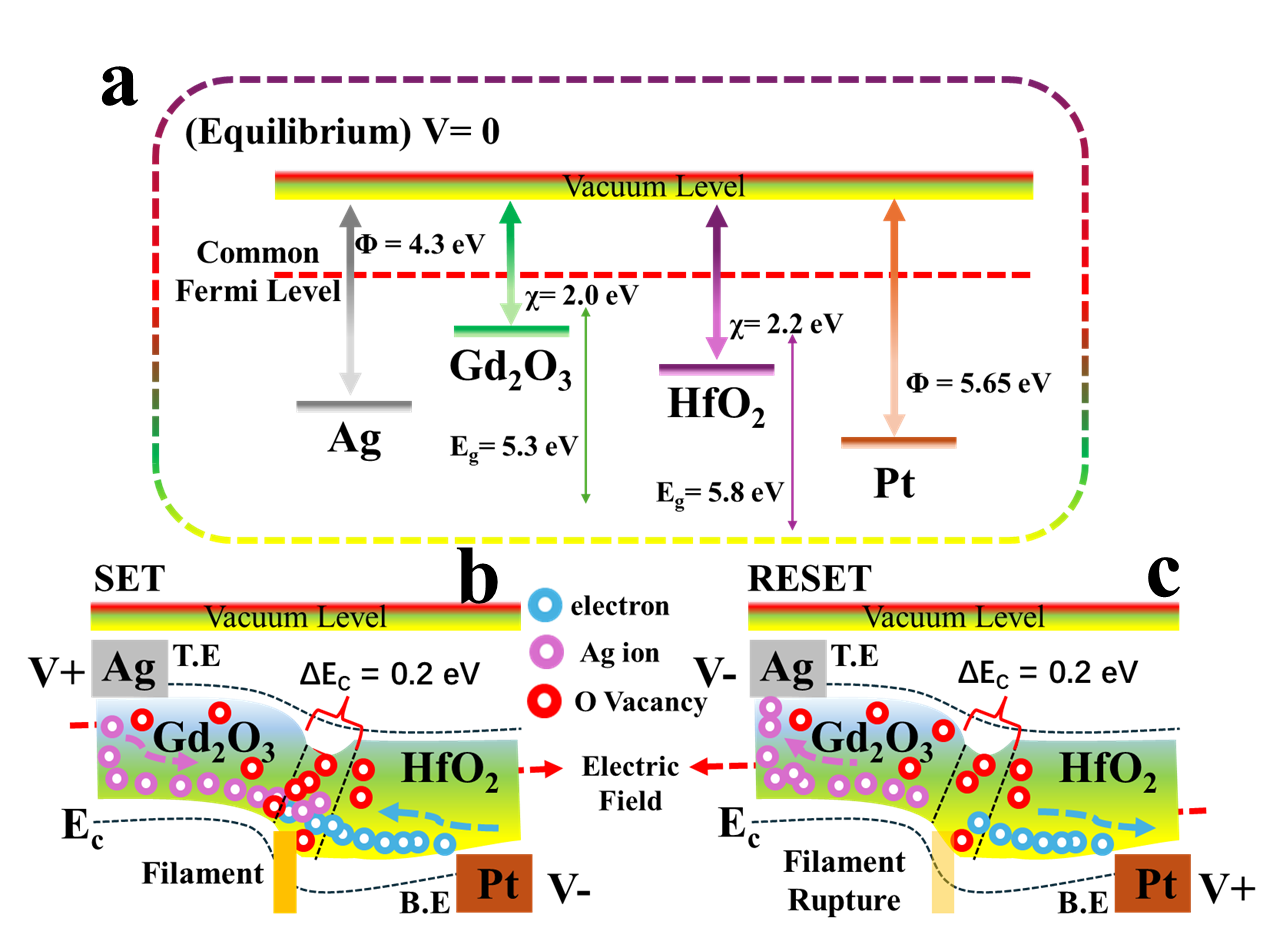
**

**Figure S1:** Energy band schematic of the bilayer memristor. (a) Energy band alignment at equilibrium of the Ag/Gd₂O₃/HfO₂/Pt bilayer memristor structure referenced to the vacuum level. The Fermi level (red dashed line) is aligned across all layers, while the conduction band minimum (CBM) and valence band maximum (VBM) reflect band bending at the interfaces. The vacuum-level-referenced electron affinities used are: χ-Gd_2_O_3_ = 2.0 eV, χ-HfO₂ = 2.2 eV, with band gaps of ~5.4 eV and ~5.8 eV, respectively. The work functions are 4.26 eV for Ag and 5.65 eV for Pt. The resulting energy offsets across the interfaces are critical for filament confinement and synaptic switching behavior.

(b) Schematic diagram of the **SET process** under positive bias applied to Ag. Ag atoms ionize into Ag⁺ and migrate through Gd₂O₃ under the influence of the electric field (red arrow), accumulating near or within HfO₂, where they are reduced to form a conductive filament (marked in red). Electrons (blue arrows) flow from Pt to Ag through the formed filament and the conduction band of HfO₂, with ohmic and space-charge-limited conduction (SCLC) as dominant transport mechanisms. Band bending is evident across both oxides, shaped by the electric field and dielectric constants.

(c) Schematic diagram of the **RESET process** under reverse bias. The electric field direction reverses, leading to the dissolution or rupture of the Ag filament due to the migration of Ag⁺ back toward the Ag electrode. The resulting discontinuity interrupts the conductive path, reverting the device to the high resistance state (HRS). Electron conduction reverts to trap-limited SCLC, with no filament bridging the oxide layers. Band bending at the interface shifts accordingly, and interfacial band offsets again modulate carrier injection.

**
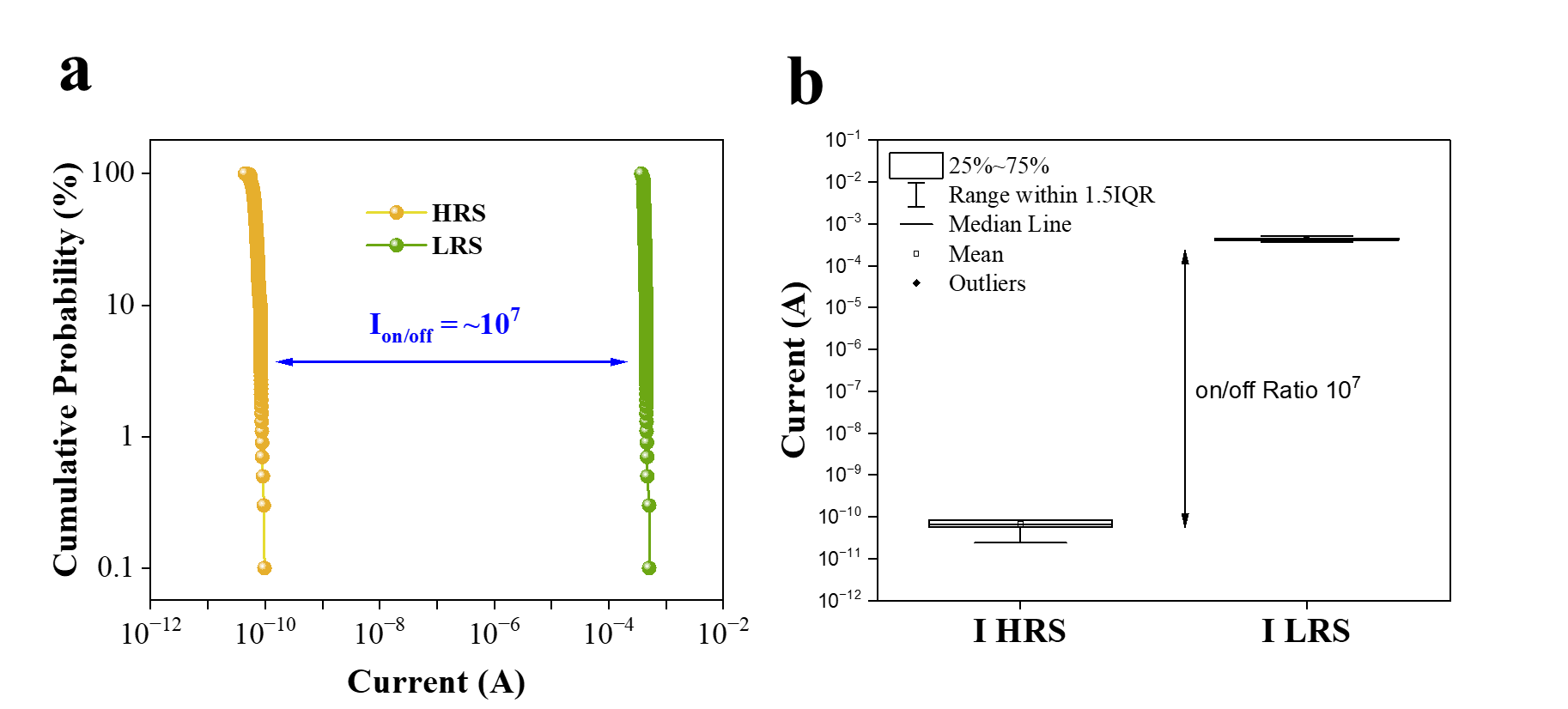
**

**Figure S2:** Single-device cycle statistics (V_read_ = 0.1 V; SET compliance = 750 µA). (a) Cumulative probability of HRS/LRS currents over 500 DC cycles; (b) Cycle-to-cycle variability analysis of the HRS and LRS current states measured over 500 consecutive DC switching cycles.

The switching uniformity and statistical reliability of the memristive device were analyzed using cumulative probability and box plot evaluations of the high-resistance state (HRS) and low-resistance state (LRS) read currents, as shown in Figure S2. These analyses quantify the consistency of resistive switching behavior under repetitive cycling, which is essential for reliable performance in neuromorphic and non-volatile memory applications. Figure S2(a) presents the cumulative probability distributions of HRS and LRS read currents over 500 consecutive cycles at a read voltage of +0.1 V. The two distributions exhibit clear separation across the entire measurement range, with an ON/OFF current ratio of approximately 10^7^. The absence of distributional overlap confirms stable and reproducible switching characteristics, indicating a well-defined memory window capable of supporting low-error readout and deterministic binary state assignment in dense crossbar architectures. Figure S2(b) shows a box plot that further quantifies the variability of the resistance states. The HRS exhibits a mean read current of 6.73 × 10^-11^ A with a standard deviation of 1.73 × 10^-11^ A, yielding a CoV = 0.204, while the LRS shows a mean of 4.19 × 10⁻⁴ A and a standard deviation of 2.70 × 10⁻⁵ A, resulting in a lower CoV = 0.0645. The lower CoV in the LRS reflects reduced statistical dispersion and improved repeatability in the conductive state, which is essential for reliable synaptic weight modulation. The narrow interquartile ranges for both HRS and LRS indicate minimal fluctuation across cycles, confirming the endurance and operational stability of the device.

**Equations**

**Mean Current**

$$\mu=\frac{1}{n}\sum_{i=1}^{n} I_{i}$$

**Standard Deviation**

$$\sigma= \sqrt{\frac{1}{n-1}\sum_{i=1}^{n} (I_{i}-\mu)^{2}}$$

**Coefficient of Variation**

$$CoV=\frac{\sigma}{\mu}$$

**ON/OFF Ratio**

$$ON/OFF Ratio=\frac{\mu_{LRS}}{\mu_{HRS}}$$

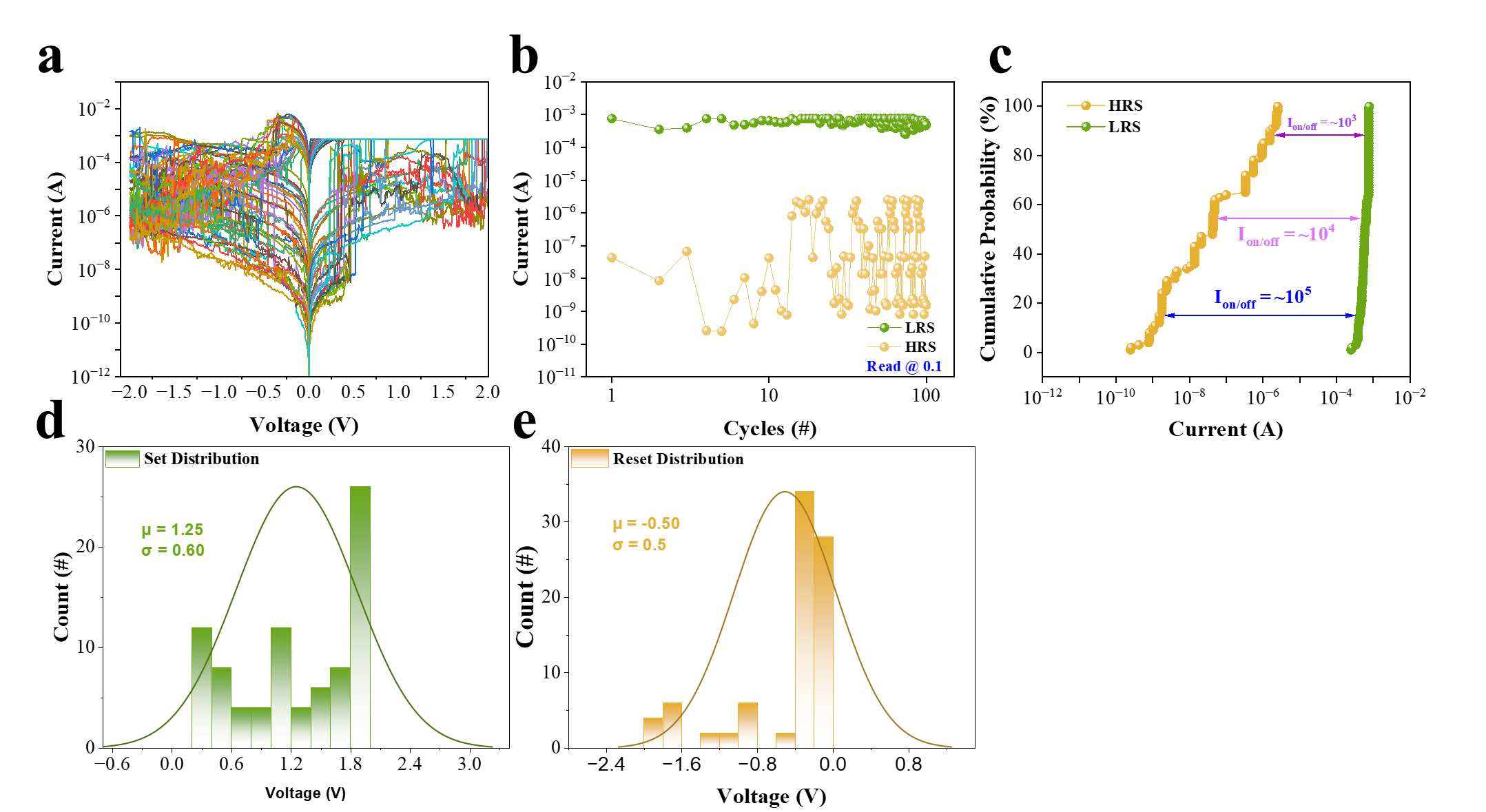


**Figure S3:** DC electrical measurements of the Ag/Gd_2_O_3_/HfO_2_/Pt device before annealing. **(a)** Multiple DC I-V switching cycles, demonstrating reproducible bipolar resistive switching in the pristine device. **(b)** Endurance characteristics showing LRS and HRS read currents at 0.1 V over 100 consecutive cycles. **(c)** Cumulative probability distribution of LRS and HRS currents at 0.1 V read voltage, showing an ON/OFF ratio of (10^5^, 10^4^, 10^3^). **(d)** Gaussian fitted SET voltage distribution (μ = 1.25, σ = 0.80).  **(e)** Gaussian fitted RESET voltage distribution (μ = -0.80, σ = 0.60).


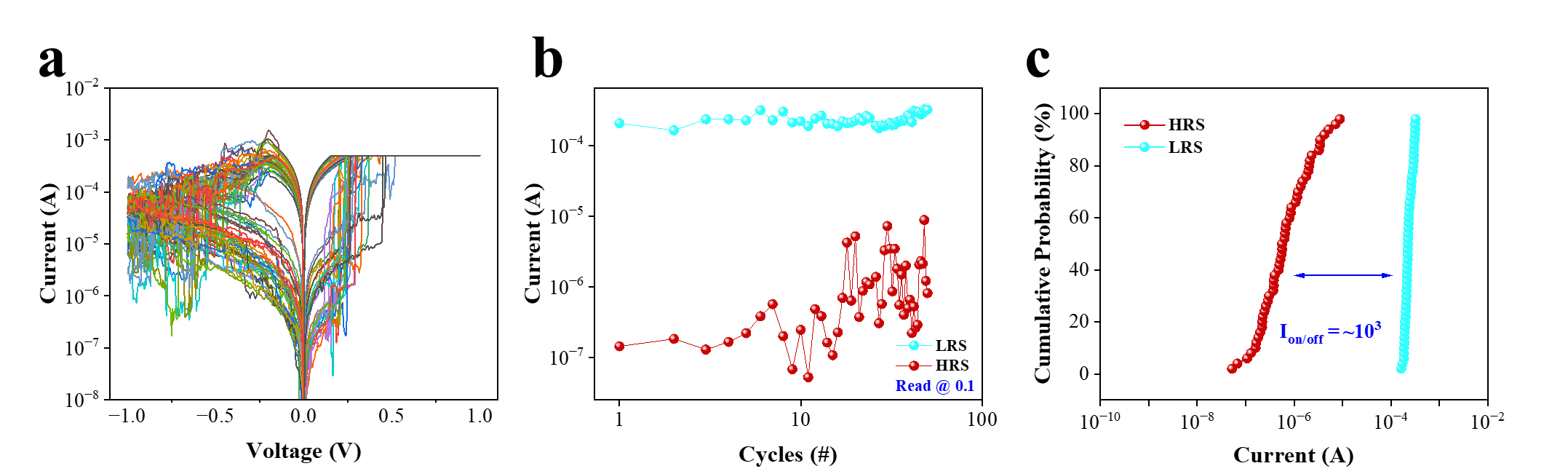


**Figure S4:** DC electrical measurements of Ag/HfO_2_/Pt single-layer memristor device. **(a)** Multiple DC I-V cycles, demonstrating the resistive switching behavior of the single-layer device. **(b)** Endurance characteristics of HRS and LRS at a read voltage of 0.1 V, plotted over repeated cycles. **(c)** Cumulative probability distribution of LRS and HRS currents at a read voltage of 0.1 V, showing an ON/OFF ratio of 10^3^.


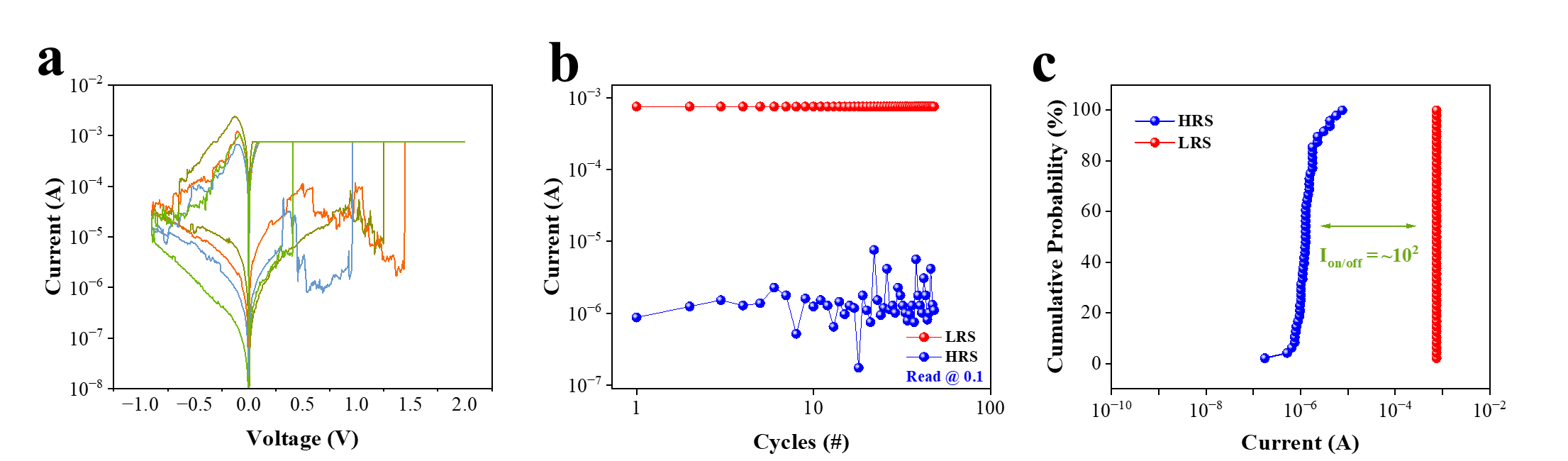


**Figure S5:** DC electrical measurements of Ag/Gd_2_O_3_/Pt single-layer memristor device. **(a)** Multiple DC I-V cycles, demonstrating the resistive switching behavior of the single-layer device. **(b)** Endurance characteristics of HRS and LRS at a read voltage of 0.1 V, plotted over repeated cycles. **(c)** Cumulative probability distribution of LRS and HRS currents at a read voltage of 0.1 V, showing an ON/OFF ratio of 10^2^.


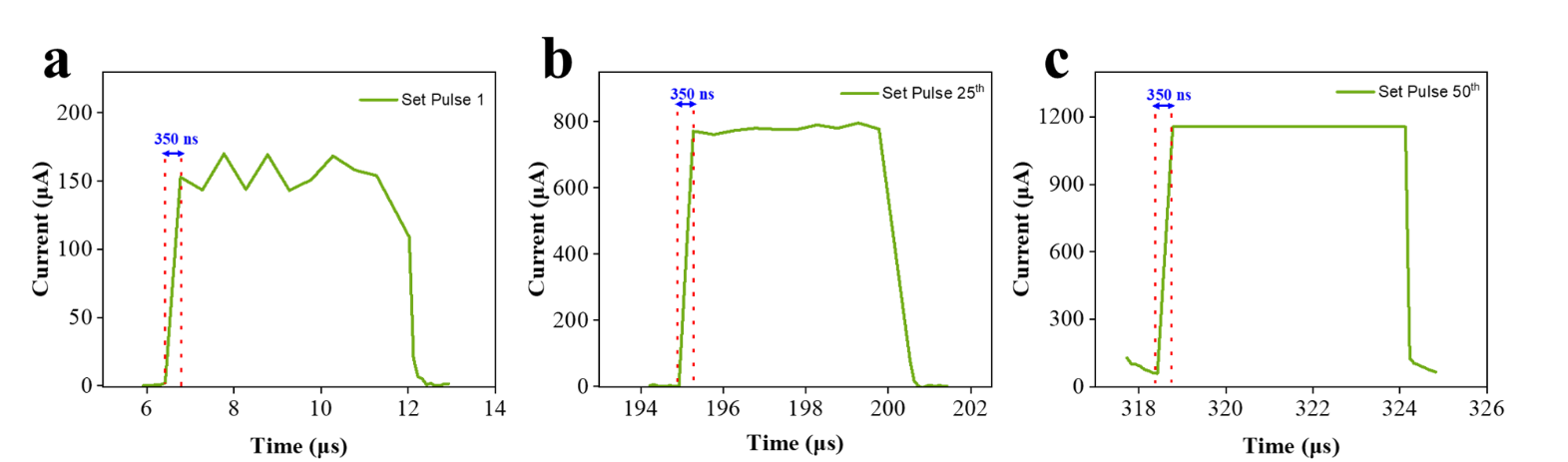


**Figure S6:** Pulse switching time analysis of the Ag/Gd₂O₃/HfO₂/Pt memristor device. **(a–c)** Device current response during the 1st, 25th, and 50th SET voltage pulses, respectively. The switching time (t_switch_) is defined as the interval for the current to rise from its initial value (I_start_) to 90% of the total current swing (I_90%_​), as indicated by the red dashed lines. Calculated switching times were 350 ns (1st pulse), 330 ns (25th pulse), and 340 ns (50th pulse), confirming consistently fast switching dynamics across repeated cycles.

Switching time is calculated by a method based on the time required for the current to increase from its initial value to 90% of the total swing.

$$t_{switch}=t_{90\%}- t_{start}$$

The equation for calculating 90% of the current is given by

$$I_{90\%}=I_{Start}+0.9( I_{final}- I_{Start})$$

**For 1^st^ set pulse**

$$I_{Start}=2.147 \times{10}^{-6} A$$

$$I_{final}=1.528 \times{10}^{-4} A$$

$$I_{90\%}=2.147 \times{10}^{-6}+0.9\left( 1.528 \times{10}^{-4}- 2.147 \times{10}^{-6} \right)$$

$I_{90\%}$=$2.147 \times{10}^{-4}A$

**Switching time**

$$t_{start}=6.425 \mu s$$

$$at I_{90\%}timet_{90\%}=6.775 \mu s$$

$$t_{switch}=6.775 \mu s- 6.425 \mu s= 0.35 \mu s=350 ns$$

Similarly, for the 25^th^ and 50^th^ pulses, switching times were calculated. Furthermore, the switching energy was calculated using the average current method.

$$E=V \cdot I_{avg}\cdot t_{switch}$$

$$V= 0.5 V$$

$$\Delta t=350 ns$$

$I_{avg}= \frac{I_{start}+ I_{final}}{2}= \frac{2.147 \times{10}^{-6}+ 1.528 \times{10}^{-4}}{2}= 7.747 \times{10}^{-5}A$

$$E=0.5\cdot7.747 \times{10}^{-5}\cdot3.5 \times{10}^{-7}=1.356 \times{10}^{-11}=13.56 pJ$$

Pulse-energy estimation. For each SET event, we compute the switching-window energy $E_{\text{switch}}=V_{\text{pulse}}\cdot I_{\text{avg}}\cdot t_{\text{switch}}$, where $t_{\text{switch}}$ is defined by the current reaching 90% of its final level and $I_{\text{avg}}$ is the average current over $t_{\text{switch}}$. This procedure isolates the energy associated with the actual state transition from post-transition dissipation, in line with prior high-speed memristor analyses that separate ‘switching’ and ‘excess’ energy[1].


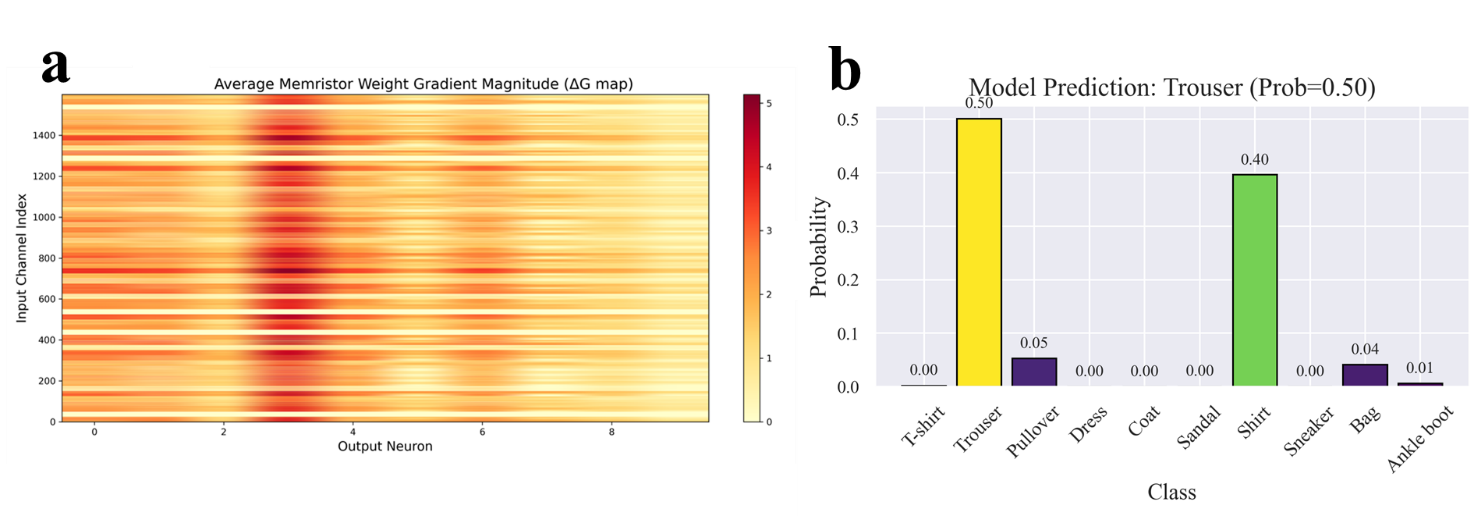
**Figure S7:** Memristor-constrained neural network learning dynamics and model prediction. **(a)** Average memristor weight gradient (ΔG map) across all input-output connections, visualizing learned feature importance and the distribution of dynamic weight updates during network training. **(b)** Model prediction output for a Fashion-MNIST test sample, showing class probabilities with the highest likelihood assigned to "Trouser" (probability = 0.50), highlighting the inference decision made by the hardware-constrained neural network. Such weight-update maps and class probability outputs provide interpretability of feature learning and enable quantification of model confidence under memristor hardware constraints.

**
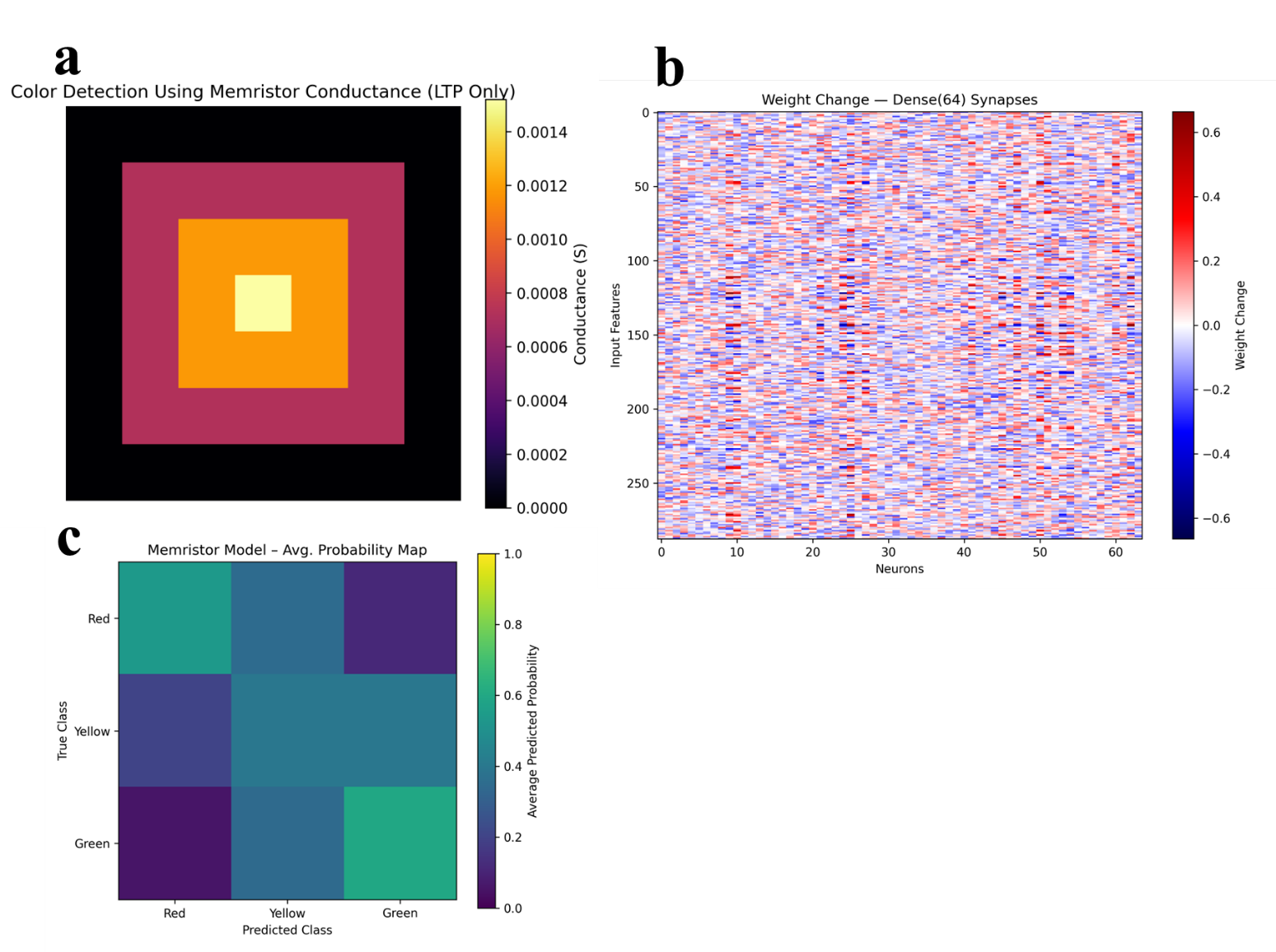
**

**Figure S8**: Model interpretability and synaptic adaptation in memristor-based color recognition. **(a)** Input color map encoded as a spatial conductance distribution (LTP only), visualizing the analog input features used by the network. **(b)** Synaptic weight change heatmap for the Dense (64) layer, illustrating distributed weight adaptation during training in response to noisy, hardware-derived conductance inputs. **(c)** Average predicted class probability map for the memristor model, revealing class-wise uncertainty and misclassification trends due to analog variability.

These analyses highlight the effects of memristor device noise on neural network decision boundaries and demonstrate the network’s capacity for adaptive learning under hardware constraints.


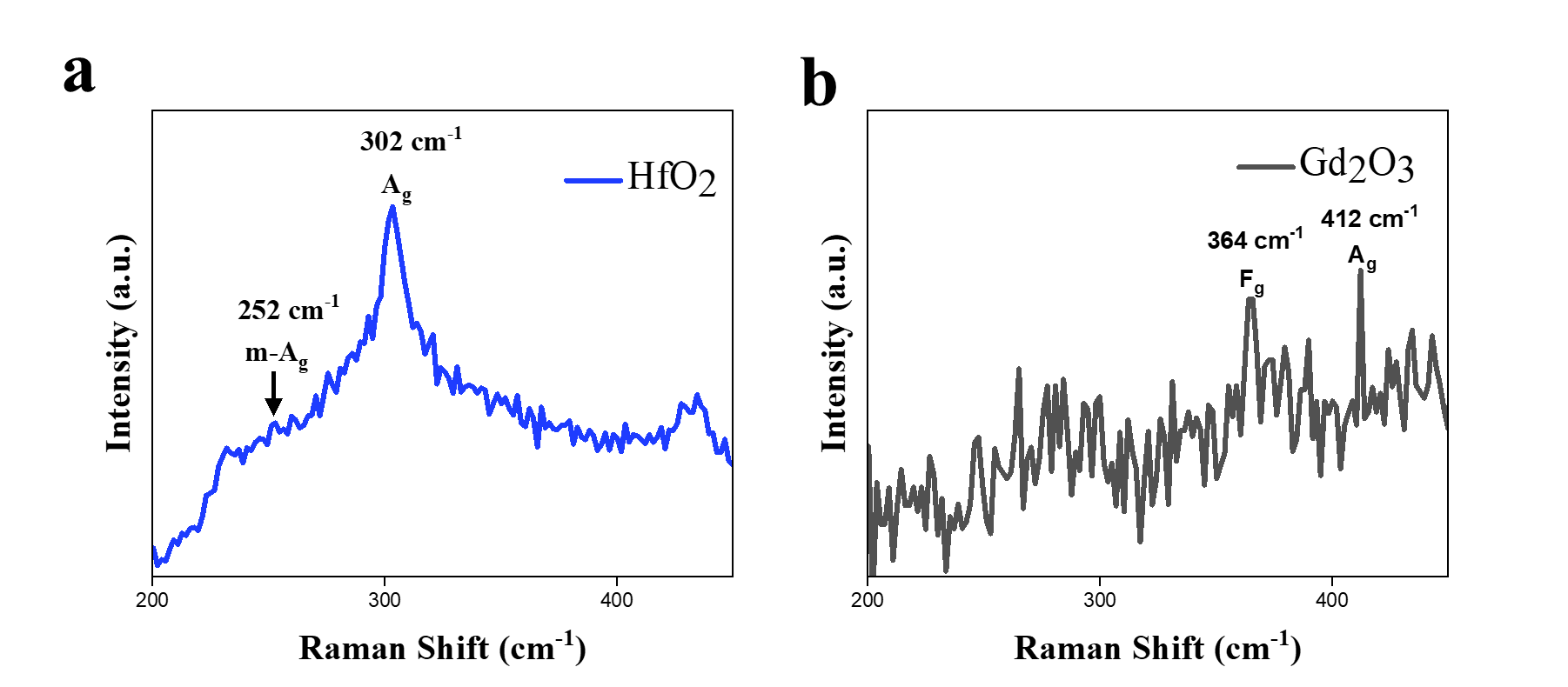


**Figure S9**: Raman spectra of oxide layers. (a) ALD-grown HfO_2_ (28 nm) thin-film shows bands at ~ 252 cm^-1^ and ~ 302 cm^-1^. A strong band at ~301 cm⁻¹ is a well-known monoclinic HfO₂ mode reported for thin films [2, 3], while a weak band near ~252 cm⁻¹ has been explicitly observed in monoclinic-rich HfO_2_ and grows with laser power/ordering[4]. (b) Sputtered-grown Gd_2_O_3_ (35 nm) thin-film exhibits peaks at ~ 364 cm^-1^ and ~ 412 cm^-1^, in agreement with the cubic (bixbyite, Ia-3) Gd_2_O_3_ fingerprint, for which comparative measurements list prominent lines at ~361, 414, 444, 569 cm^-1^[5-7].

| **Parameter** | **Before Heat Treatment** | **After Heat Treatment** |
| --- | --- | --- |
| **Minimum Height (nm)** | −6.035 | −5.658 |
| **Maximum Height (nm)** | 21.217 | 12.290 |
| **Mid Height (nm)** | 7.591 | 3.316 |
| **Mean Height (nm)** | 0.047 | 0.000 |
| **Peak-to-Valley (Rpv, nm)** | 27.252 | 17.947 |
| **Root Mean Square (Rq, nm)** | 1.759 | 1.695 |
| **Average Roughness (Ra, nm)** | 1.318 | 1.233 |
| **Ten-point Height (Rz, nm)** | 26.063 | 16.273 |
| **Skewness (Rsk)** | −1.379 | −0.443 |
| **Kurtosis (Rku)** | 11.670 | 3.673 |

**Table 1**: Surface roughness parameters of the Ag/Gd₂O₃/HfO₂/Pt device before and after heat treatment. Quantitative atomic force microscopy (AFM) analysis comparing surface morphology before and after annealing. The post-annealing surface exhibits reduced peak-to-valley height (Rpv), mid and mean height, and average roughness (Ra), along with decreased skewness (Rsk) and kurtosis (Rku), indicating smoother, more uniform film morphology after thermal treatment.

| **Element / Orbital** | **Observed Binding Energy (eV)** | **Chemical State / Assignment** |
| --- | --- | --- |
| **Hf 4f_7/2_ (HfO₂)** | 16.8 | Hf ^4+^ in fully oxidized HfO₂ confirms the formation of stoichiometric hafnium oxide. |
| **Hf 4f_5/2_ (HfO₂)** | 18.6 | Spin-orbit partner confirming Hf ^4+^ in HfO₂. |
| **Hf 4f (HfOₓ sub peak)** | (near 17–18 eV) | Assigned to HfO_x_ (non-stoichiometric) indicates oxygen-deficient Hf regions. |
| **O 1s in HfO₂ O_L_** | 529.6 | Lattice oxygen (O^2-^) confirms strong metal-oxygen bonding in HfO₂. |
| **O 1s in HfO₂ O_V_** | 532.2 | Oxygen vacancies contribute to enhanced resistive switching. |
| **Gd 4d_5/2_** | 145.5 | Gd³⁺ oxidation state confirms formation of Gd₂O₃ as an insulating layer. |
| **Gd 4d_3/2_** | 141.0 | Spin–orbit pair of Gd 4d further supports Gd₂O₃ formation. |
| **O 1s in Gd₂O₃ O_L_** | 529.5 | Lattice oxygen in Gd₂O₃ indicates good oxide formation. |
| **O 1s in Gd₂O₃ O_V_** | 531.5 | Oxygen vacancies in Gd₂O₃ enhance conduction pathways in switching devices. |

**Table 2:** XPS binding energies and chemical state assignments for HfO₂ and Gd₂O₃ thin films.
Summary of observed binding energies and corresponding chemical states for key elements in HfO₂ and Gd₂O₃ layers. The presence of fully oxidized (Hf^4+^, Gd^3+^), non-stoichiometric HfOx, lattice oxygen (O_L_), and oxygen vacancies (O_V_) is confirmed, indicating successful oxide formation and the presence of defects critical for resistive switching.

| **Device Type** | **Dataset** | **Ideal Accuracy (%)** | **Accuracy (%)** | **Mapping Approach** | **Reference** |
| --- | --- | --- | --- | --- | --- |
| Ag/Gd_2_O_3_/HfO_2_/Pt | F-MNIST | 89% | 78% | Pulse-driven exponential mapping based on measured LTP/LTD characteristics; synaptic weights derived without additional normalization, linearization, or compensation | **This Work** |
| Ag/ZnO_x_/FTO | F-MNIST | ~85% | ~79% | Linearized LTP/LTD characteristics are employed to improve weight update uniformity | [8] |
| Ag/NbOx/ZrO_2_/Pt | F-MNIST | ~98% | ~80% | Multilevel conductance states utilized with partial linearization and state quantization | [9] |
| Pt/Na: TiO_2_/Pt | F-MNIST | ~89% | ~80% | Conductance-to-weight mapping with implicit normalization and stabilized switching behavior | [10] |
| Al/TiO_x_/Al | F-MNIST | -- | 82% | Hardware-in-the-loop (in-situ) training with adaptive learning rate and compensation of device non-idealities | [11] |

**Table 3:** Comparison of Fashion-MNIST (F-MNIST) classification performance in memristor-based neuromorphic systems, illustrating how different weight-mapping strategies based on experimentally measured device characteristics influence the obtained accuracy.

**References**

1. Teja Nibhanupudi, S.; Roy, A.; Veksler, D.; Coupin, M.; Matthews, K. C.; Disiena, M.; Ansh; Singh, J. V.; Gearba-Dolocan, I. R.; Warner, J., *Nature Communications* **2024,** *15* (1), 2334.DOI <https://doi.org/10.1038/s41467-024-46372-y>

2. Salas-Rodríguez, S.; González-Moreno, F. I.; Woo-García, R. M.; Herrera-May, A. L.; López-Huerta, F.; Caballero-Briones, F., *Applied Sciences* **2025,** *15* (3), 1573.DOI <https://doi.org/10.3390/app15031573>

3. Li, S.; Zhang, Y.; Yang, D.; Yang, W.; Chen, X.; Zhao, H.; Hou, J.; Yang, P., *Physica B: Condensed Matter* **2020,** *584*, 412065. DOI <https://doi.org/10.1016/j.physb.2020.412065>.

4. Ivanov, Y. D.; Malsagova, K. A.; Popov, V. P.; Kupriyanov, I. N.; Pleshakova, T. O.; Galiullin, R. A.; Ziborov, V. S.; Dolgoborodov, A. Y.; Petrov, O. F.; Miakonkikh, A. V.; Rudenko, K. V.; Glukhov, A. V.; Smirnov, A. Y.; Usachev, D. Y.; Gadzhieva, O. A.; Bashiryan, B. A.; Shimansky, V. N.; Enikeev, D. V.; Potoldykova, N. V.; Archakov, A. I., *Molecules* **2021,** *26* (12). DOI 10.3390/molecules26123715.

5. Manohara, B., *Rare Earth Activated Gedelonium Oxide Nanophosphors*. Nitya Publications: **2020**.PP(1-44)

6. Vijayaprasath, G.; Habibulla, I.; Dharuman, V.; Balasubramanian, S.; Ganesan, R., *ACS Omega* **2020,** *5* (29), 17892-17899. DOI 10.1021/acsomega.9b04284.

7. Le Luyer, C.; García‐Murillo, A.; Bernstein, E.; Mugnier, J., *Journal of Raman Spectroscopy* **2003,** *34* (3), 234-239.DOI <https://doi.org/10.1002/jrs.980>

8. Gao, K.; Sun, B.; Yan, W.; Yang, B.; Cao, Z.; Cui, Y.; Wang, M.; Luo, S.; Chen, X.; Shao, J., *Materials Today Chemistry* **2025,** *47*, 102841. DOI <https://doi.org/10.1016/j.mtchem.2025.102841>.

9. Zulfiqar, M. W.; Nisar, S.; Zulfiqar, R.; Ahmad, M.; Rabeel, M.; Ghazanfar, H.; Wabaidur, S. M.; Rasheed, A.; Kim, H.; Dastgeer, G., *Journal of Materials Chemistry C* **2026**.DOI <https://doi.org/10.1039/D5TC04061B>

10. Kim, M.; Lee, S.; Kim, S. J.; Lim, B. M.; Kang, B.-S.; Lee, H.-S., *ACS Applied Materials & Interfaces* **2024,** *16* (13), 16453-16461. DOI 10.1021/acsami.3c19531.

11. Jang, J.; Gi, S.; Yeo, I.; Choi, S.; Jang, S.; Ham, S.; Lee, B.; Wang, G., *Advanced Science* **2022,** *9* (22), 2201117. DOI <https://doi.org/10.1002/advs.202201117>.
